# Functionally Graded Polymer Lattice Structures Enabled by Multi-Material Fused Deposition Modeling: Current Progress, Challenges, and Future Perspectives

**DOI:** 10.3390/polym18202483

**Published:** 2026-10-11

**Authors:** Abdullahi Idris Usman, Muhammet Muaz Yalçın

**Affiliations:** Mechanical Engineering Department, Sakarya University, 54050 Sakarya, Turkey; abdullahi.usman@ogr.sakarya.edu.tr

**Keywords:** multi-material, FDM, functionally graded lattice, polymer metamaterials, energy absorption, TPMS, functional grading strategies

## Abstract

Functionally graded lattice (FGL) structures can decouple stiffness, strength and energy absorption. Multi-material fused deposition modeling (MM-FDM) is an accessible route to lattices that vary in composition and topology within one print. This review examines MM-FDM polymer FGLs through a PRISMA 2020-informed screening of 610 records. Of the 126 screened references, 65 primary studies meet all eligibility criteria and form the core evidence, and 20 are supporting studies. Four grading strategies were identified: material-based zoning, geometry-induced grading, combined material–geometry grading and process-induced grading, applied mainly to strut-based, triply periodic minimal surface (TPMS) and hybrid plate–TPMS architectures. Pairing rigid thermoplastics (PLA, ABS, PETG) with hyperelastic TPU increased specific energy absorption by up to two- to threefold over single-material lattices, but the gain over the rigid constituent alone was smaller and, in several quasi-static studies, negative; it depends on configuration, baseline and loading rate. Graded lattices collapse layer by layer, yet their energy gain over uniform lattices of equal mass is often modest. TPMS architectures are reported to reduce nodal stress concentrations. Most evidence remains at the specimen level. The main limitations are weak interfaces between incompatible polymers, process-induced defects, models that neglect manufacturing effects and the lack of standardized test protocols.

## 1. Introduction

### 1.1. Functionally Graded Materials: Concept and Evolution

Historically, the search for engineering materials with contradictory mechanical properties, i.e., both high strength and ductility, or both stiffness and toughness, has been limited by the homogeneous nature of traditional engineering materials [1]. The development of functionally graded materials (FGMs) provided an innovative way to overcome this limitation. FGMs are materials whose composition, microstructure, or porosity vary across space, leading to controlled gradients in properties throughout the body of a structural part [2,3]. The principle of FGMs was inspired fundamentally by nature; numerous examples of naturally occurring, biologically optimized structures, such as trabecular bone, bamboo, and fish scales, exhibit optimal gradients that maximize load-bearing while minimizing mass [2,4,5]. The archetypal biological example is bone, whose stiff cortical shell and porous trabecular core together minimize peak stress and maximize impact energy absorption [2,6]. Similarly, Wen and Li [7] demonstrated that gradient profiles similar to those found in bamboo significantly improve compressive energy absorption in bionic FGM cubic and honeycomb FDM lattice structures.

After being used primarily to mitigate thermal stresses in aerospace thermal barrier coatings [8], FGMs have increasingly been applied in other fields, including biomedical implants, automotive crashworthiness, and soft robotics [1,9,10]. In general, FGMs may be divided into compositional gradations, in which the materials that make up the structure vary continuously with respect to one another, and microstructural gradations, in which geometric features such as pores and grains vary continuously [2,9]. A major distinction in the history of this technology has been the shift away from layer-to-layer transitions toward continuous grading. Continuous grading eliminates the discontinuous interfaces present at each layer in layered composites, which typically act as failure locations for delamination [3,11].

### 1.2. Architected Lattices as Functional Grading Platforms

**Figure 1:**
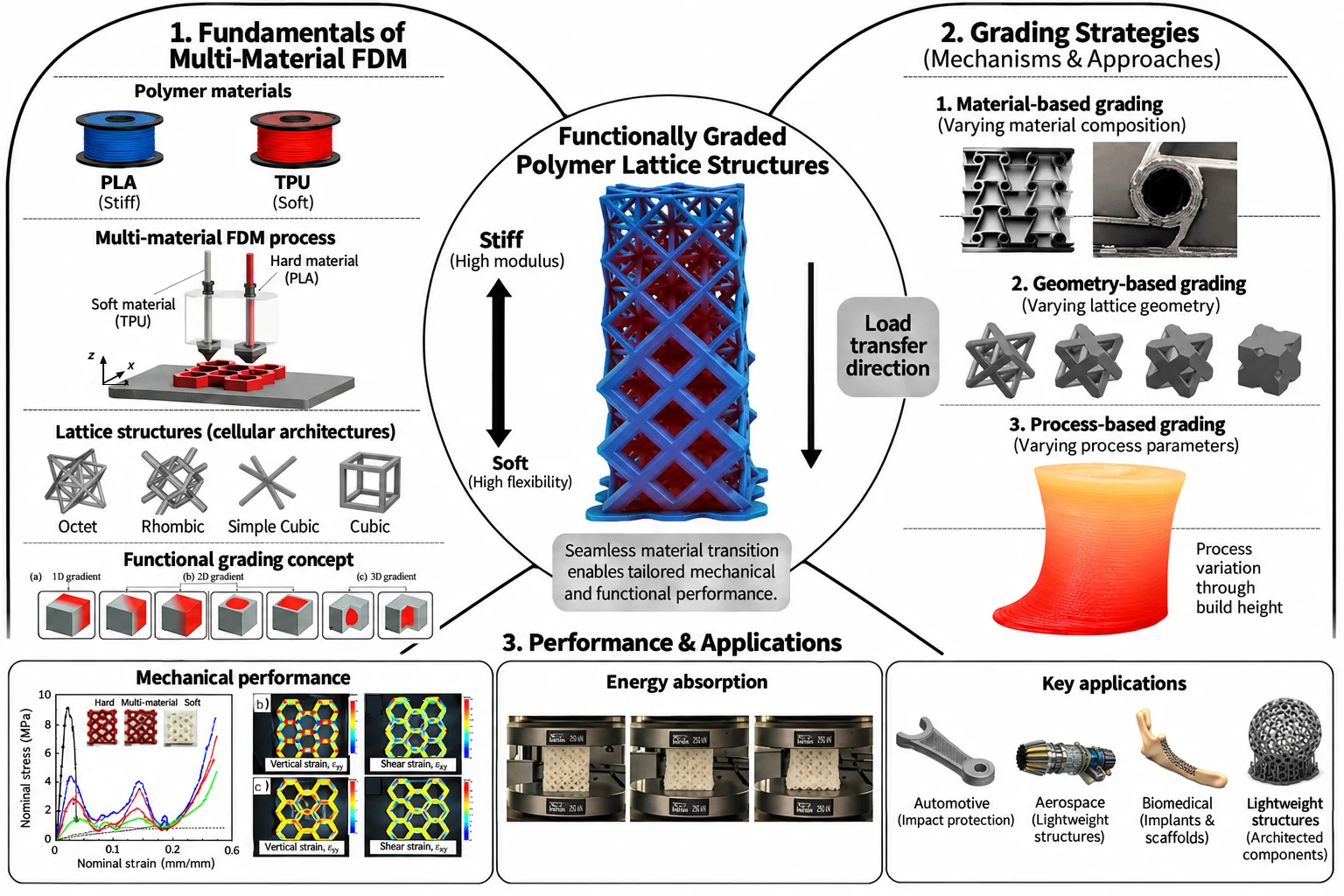
An illustrative representation of functionally graded polymer lattice structures produced by multi-material fused deposition modeling.

Research on the structural mechanics of cellular solids has shown that cellular solids, especially architected lattice structures, provide the most significant geometrical basis for achieving functional grading in solid mechanics [2]. Compared with stochastic foam structures, lattice structures can be constructed using periodic tessellations of unit cells, providing the designer with a means to systematically control response characteristics [12,13]. Strut-based lattices (body-centered cubic, octet) and surface-based lattices (triply periodic minimal surfaces, TPMS) are common architectural forms of cellular solids. TPMS architectures, including Gyroid and Schwarz Primitive types, have become more prominent due to their mathematically continuous surfaces; they can support themselves during fabrication and provide better fatigue life than strut-nodal designs [14,15]. Figure 1 presents a representative example of such functionally graded lattice architectures realized through multi-material FDM.

Spatial variations in the degree of grading in lattices are achieved by varying the relative density of the material, the cell size, or the topology [2]. Geometric grading allows designers to modulate local stiffness by varying strut thickness or void size, thereby shifting the failure mechanism from catastrophic shear failure to progressive layer-by-layer collapse, as analyzed in Section 6.2 [2]. Graded lattices consequently exhibit greater SEA and plateau stress than ungraded counterparts (see Section 6.2) [2]. Nevertheless, geometric grading alone has a limitation: lowering the relative density to save mass also increases compliance and lowers strength. Stiffness, strength and mass therefore remain coupled, and geometric manipulation alone can resolve this trade-off only partially [6].

### 1.3. FDM for Polymer Functionally Graded Structures

Additive manufacturing (AM) has enabled the production of these complex architectural gradient shapes. Fused deposition modeling (FDM), a material extrusion (MEX) process, is currently the most commonly used AM technology for producing polymeric structures due to its low cost, flexibility in material selection, and availability [16,17]. FDM (originally a trade name) and fused filament fabrication (FFF) both denote filament-fed thermoplastic MEX, and fused granular fabrication (FGF) denotes its pellet-fed variant. In this review, FDM is used as the general term for thermoplastic MEX because most of the reviewed studies use it; FFF and FGF are used only where a cited study refers specifically to filament- or pellet-fed systems. MEX denotes the wider process category, which also includes paste and ink extrusion, and is therefore not used as a synonym for FDM. MM-FDM denotes FDM in which two or more thermoplastic feedstocks are deposited within one build, by multiple nozzles, tool changers, or single-nozzle mixing or co-extrusion, and MMAM refers to multi-material AM across all process families [9,18]. FDM produces structures by depositing melted thermoplastic filaments on top of each other to create a layer-by-layer structure. In addition, this enables the creation of highly detailed features, such as complex internal channels and overhanging surfaces that characterize lattice metamaterials [9].

In recent years, advances in controlling FDM processes have enabled new ways of grading, called process-driven grading. This uses dynamic changes in printing parameters, such as extruder speed and flow rate, to create variable local densities while keeping the toolpath geometry constant [19]. Although there are many advantages to this approach, single-material lattices produced via FDM are still limited by the inherent properties of the feedstock polymer. Polylactic acid (PLA) has good stiffness but lacks toughness, whereas thermoplastic polyurethane (TPU) is highly elastic but lacks load-carrying capacity [11,12]. The layer-by-layer deposition method used in FDM results in anisotropic properties and may introduce voids between layers that can reduce the overall structural integrity of graded lattices unless they are carefully optimized [20,21].

### 1.4. Role of Multi-Material FDM in Achieving True Functional Grading

One of the main reasons why multi-material FDM (MM-FDM) is important is that it enables true functional grading that combines both geometric and composition-based variations [9,11]. With MM-FDM, different types of polymers (such as rigid thermoplastics and soft elastomers) can be deposited within a single print job, resulting in independent variation in stiffness and geometry [18,22]. The most documented application of this principle is a PLA outer shell with a TPU core, which achieves SEA values 2–3 times higher than single-phase counterparts through the confinement mechanism detailed in Section 4.1 [6].

MM-FDM is generally accomplished using two nozzle heads or a single nozzle head with active mixing extruders [18,23]. Single-nozzle-head active-mixing extruders are especially attractive when continuous compositional transitions are required. They allow for seamless transitions from 0% material A to 100% material B, and vice versa. This eliminates the potential for stress concentrations caused by discrete material interfaces. One major limitation of MM-FDM is ensuring that the selected polymers bond well. Poor interfacial bonding between chemically disparate polymers may lead to debonding under external loads [11,20]; similar interfacial failures have been reported in metal–polymer multimaterial parts [24]. Therefore, the successful application of multiple material grading requires sophisticated slicing algorithms that assign materials at the voxel level and knowledge of the bonding mechanisms governing temperature and viscosity during the deposition of dissimilar polymers side by side [9].

### 1.5. Motivation, Gaps in Existing Reviews, and Objectives

To contextualize this study relative to earlier studies, we find it helpful to outline the specific area of interest. Nazir et al. [9], for example, conducted a comprehensive survey of multi-material AM techniques across all manufacturing process technologies and material groups (polymers, metals, ceramics, and composite materials), in which cellular metamaterials were treated as just one possible field of use among others. Aghajani et al. [12], likewise, focused on composite lattices but presented results across the entire spectrum of AM methods, including powder bed fusion and vat photopolymerization, with material grading being only one of multiple design features. Almesmari et al. [14] conducted a comprehensive review of polymeric metamaterials in relation to their structural and technological features, as well as various architectural strategies and AM processes, but did not specifically address functional grading as the sole organizing principle. Bhat et al. [2], in contrast, developed a framework for multifunctional lattices using different AM processes and in two distinct system types (metallic and polymeric). Likewise, Zhang et al. [25] documented variable density lattice strategies primarily for single-material contexts. Hence, none of the prior reviews focused on the combination of features that characterizes our work: (a) thermoplastic polymer feedstocks, (b) FDM as the production technology, and (c) functional grading as the common design perspective. Therefore, due to its relative narrowness of focus, we can investigate problems that would be abstracted away by larger reviews (notably the relationship between process constraints specific to FDM (e.g., nozzle switching, viscosity mismatch, layer-by-layer diffusion bonding) and the four functional grading strategies (material-based, geometry-induced, combined, and process-induced) when compared in Section 4. The structured, PRISMA 2020-informed screening and the tiered classification of the evidence (Section 2) make the evidence base of this synthesis explicit.

### 1.6. Scope and Organization of the Paper

The objective of this review is to synthesize current research on MM-FDM of functionally graded polymer lattice structures. It examines the strategies used to spatially grade material composition and geometry, and surveys which lattice architectures are most commonly employed. The review then compares mechanical and functional performance against single-material counterparts, before turning to the manufacturing constraints and research gaps that currently limit industrial translation. The scope includes both strut-based and TPMS architectures and is limited to thermoplastics with particular emphasis on rigid–hyperelastic pairings (PLA/ABS/PETG with TPU), while excluding metal FGLs, vat photopolymerization and powder-bed fusion, except where such studies are cited as adjacent studies for comparison (Supplementary Table S1).

The rest of the article is laid out as follows: In Section 2, the literature search, screening and evidence classification are described, together with a bibliometric overview of the core studies. In Section 3, the basics of MM-FDM are described, including extrusion system architectures, polymer compatibility, and process-specific manufacturing challenges. In Section 4, four functional grading approaches are compared: material-based, geometry-induced, material–geometry-combined, and process-induced. In Section 5, lattice structures developed through MM-FDM are examined, and strut-based, TPMS, and hybrid TPMS plate topologies are compared. Section 6 addresses the mechanical and functional behavior of these structures, focusing on mechanisms of energy absorption, deformation modes, and material interface effects, and concludes with design guidelines drawn from the reviewed studies (Section 6.6). Section 7 discusses application areas (energy absorption, lightweight structures, vibration damping and soft–rigid systems) and classifies each by its level of evidence. In Section 8, computer design tools, including homogenization, finite element simulations, voxel-based workflows, and data-driven optimization, are discussed and compared. In Section 9, limitations and open questions related to manufacturing are documented. In Section 10, areas requiring further research are identified, and guidance for future development is provided. Finally, in Section 11, the synthesis concludes.

## 2. Literature Search Methodology and Bibliometric Analysis

### 2.1. Search Strategy and Eligibility

Peer-reviewed literature was identified in Web of Science Core Collection, Scopus and ScienceDirect using the Boolean strings S1–S5 (Table 1), and in Scite.ai, a citation-based discovery platform, using string S1 only. All sources were searched between November 2025 and January 2026. Searches were limited to English-language research articles, review articles and conference papers (Web of Science and Scopus) published from 2020 onwards. The field settings used in each database were not recorded, so the complete set of retrieved records is provided (Supplementary Material S3). The review followed a PRISMA 2020-informed workflow [26], with the departures stated in Section 2.4.

Core studies had to meet three criteria: (i) a thermoplastic feedstock, including filled thermoplastics, processed by material extrusion (FDM, FFF, or FGF); (ii) a lattice or cellular architecture; and (iii) multi-material processing, functional grading, or both. Slicer infill was counted as a cellular architecture only when its pattern or density was studied. Studies of other processes (e.g., vat photopolymerization, powder-bed fusion) or of metallic, ceramic or thermoset systems were not eligible as core studies.

### 2.2. Screening and Selection

The searches returned 616 records, which were merged into one BibTeX file. In Stage 1, a Python script (Supplementary Material S3) scored the title, abstract and keywords of each record against five keyword dictionaries (Supplementary Table S2). Each criterion scored either 0 or its full weight: polymer material (2 points), FDM/material extrusion (2), lattice architecture (2), multi-material processing (2) and functional grading (1), giving a maximum score of 9. Records scoring 7–9 were retained (n = 34), those scoring 0–3 were excluded (n = 373), and the remaining 209 were classed as borderline (83 scoring 5–6 and 126 scoring 4). Six duplicates were then removed, leaving 610 unique records; re-running the script on these gives the same 34 retained records.

Of the 209 borderline records, 101 were carried forward to Stages 2–3 (44 scoring 5–6 and 57 scoring 4). No additional weighting was applied in Stage 2: all 44 records scoring 5–6 were retained. In Stage 3, GitHub Copilot (version 1.136) ranked 57 records, scoring 4 against the eligibility criteria and shortlisted 50; the ranking rule was not recorded.

**Figure 2:**
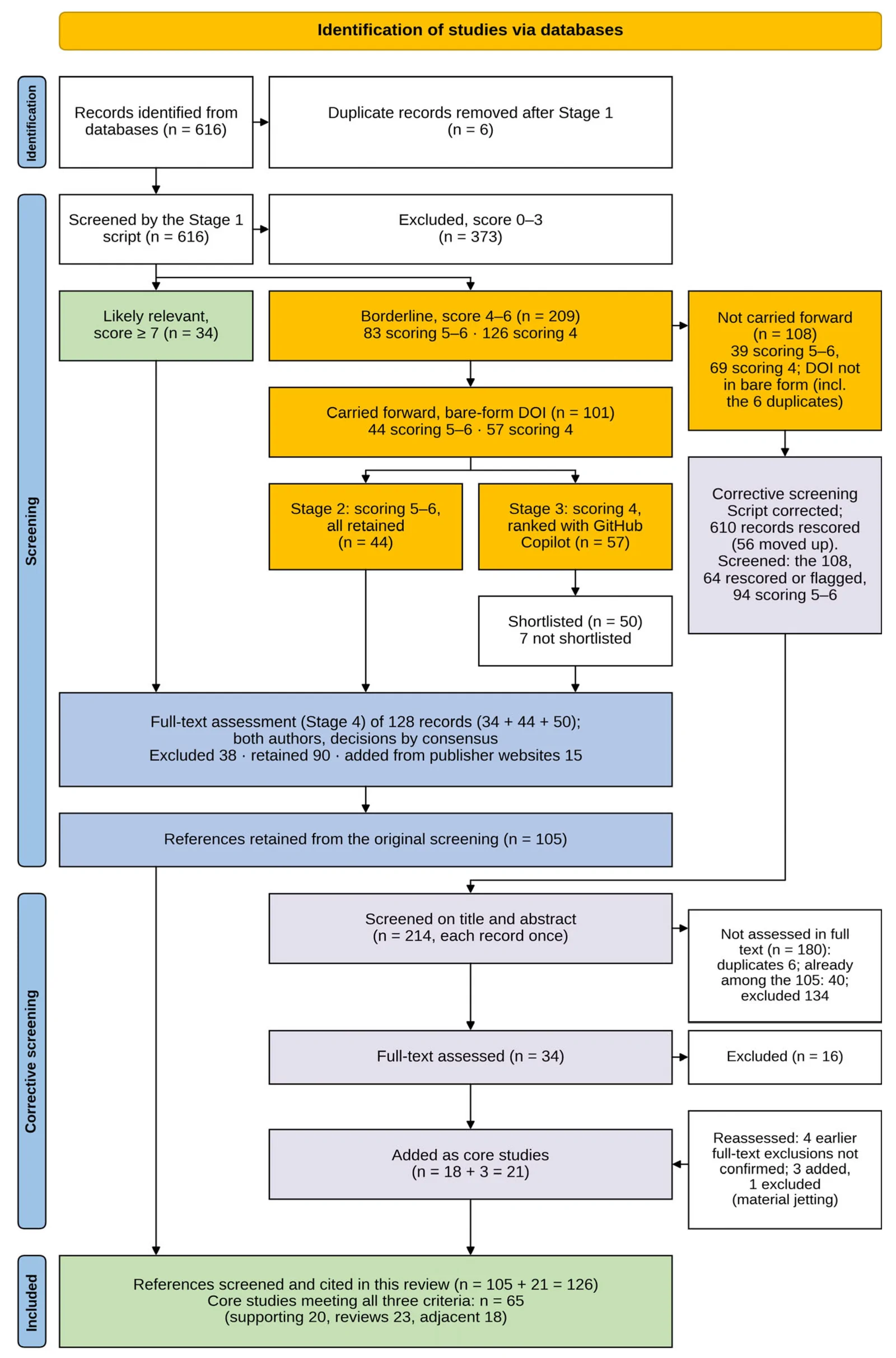
Flow diagram of the literature search and screening process, adapted from PRISMA 2020 [26]. Full-text exclusion reasons were assigned retrospectively (Supplementary Table S1); departures from PRISMA 2020 are listed in Section 2.4. Of the 209 borderline records, 101 (44 scoring 5–6 and 57 scoring 4) were carried forward to Stages 2–3; the other 108 were assessed in the corrective screening (Section 2.2), which added 21 core studies.

**Figure 3:**
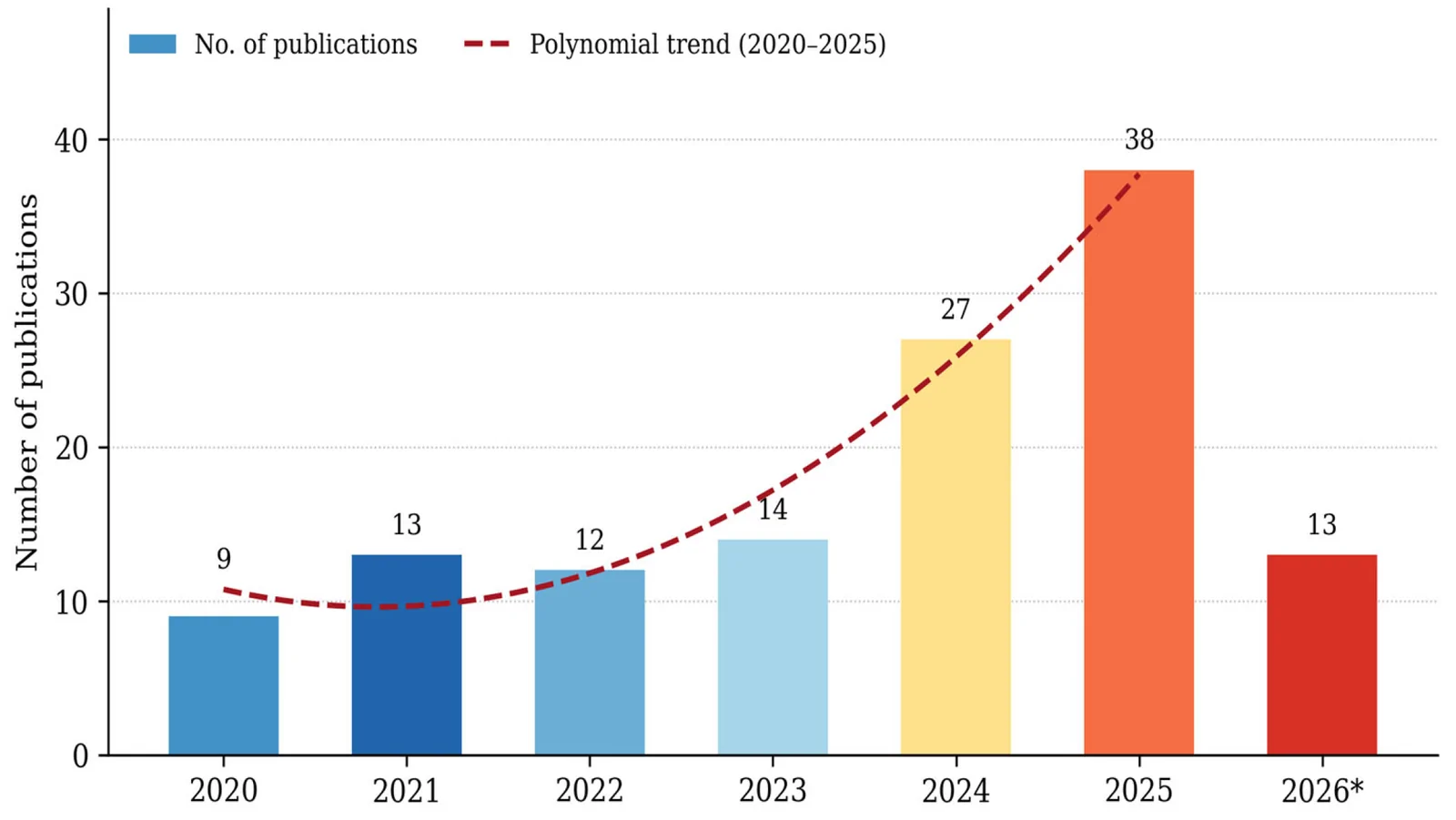
Annual number of publications among the 126 references retained after screening, including the corrective screening (2020 to January 2026). The dashed line is a second-order polynomial trend fitted to the complete years 2020–2025. * 2026 covers records retrieved up to January 2026.

Both authors assessed the resulting 128 records (34 + 44 + 50) in full text together and made every decision by consensus, so screening was not independent. Ninety records were retained and 38 were excluded; exclusion reasons were not recorded at the time and were assigned retrospectively (Figure 2). During full-text reading, both authors also searched publisher websites (Springer, Wiley, MDPI, ASME, Taylor & Francis) and added 15 records: 13 had been retrieved by the database searches but had been removed by the automated stages, and 2 had not been retrieved. This gave 105 records. A later check showed that the Stage 1 script read each BibTeX field only up to its first internal brace, so many titles had been scored in truncated form. A corrective screening was therefore carried out. The script was corrected and the 610 records were rescored; 56 records moved to a higher category and none to a lower one. The 108 borderline records that had not been carried forward, the rescored records, 13 records whose titles indicated possible eligibility despite a score below 4, and all records scoring 5–6 after rescoring were then screened on title and abstract, with a decision and reason recorded for each (Supplementary Table S1; Supplementary Material S3). Thirty-four records were assessed in full text, and 18 met all criteria and were added as core studies; two of them had been excluded at the full-text stage of the original screening. The four other full-text exclusions whose eligibility had not been confirmed were then reassessed: three met all criteria and were added as core studies, and one was excluded because it used material jetting. The corrective screening was carried out, and each decision was recorded with its reason and checked against the full text or, where the full text was not accessible, the abstract, and the authors reviewed every full-text-stage decision that rested on a judgment call or on the abstract alone, as well as the inclusion of a study co-authored by one of the authors. Figure 2 shows the full flow. Supplementary Table S1 gives the score and decision for every record and the role of each of the 126 references in this review (105 from the original screening and 21 from the corrective screening); Figure 3 shows all screened references, and only the 65 references that meet all three criteria (core studies, identified in Supplementary Table S1) are counted inFigure 4.

### 2.3. Bibliometric Analysis

**Figure 4:**
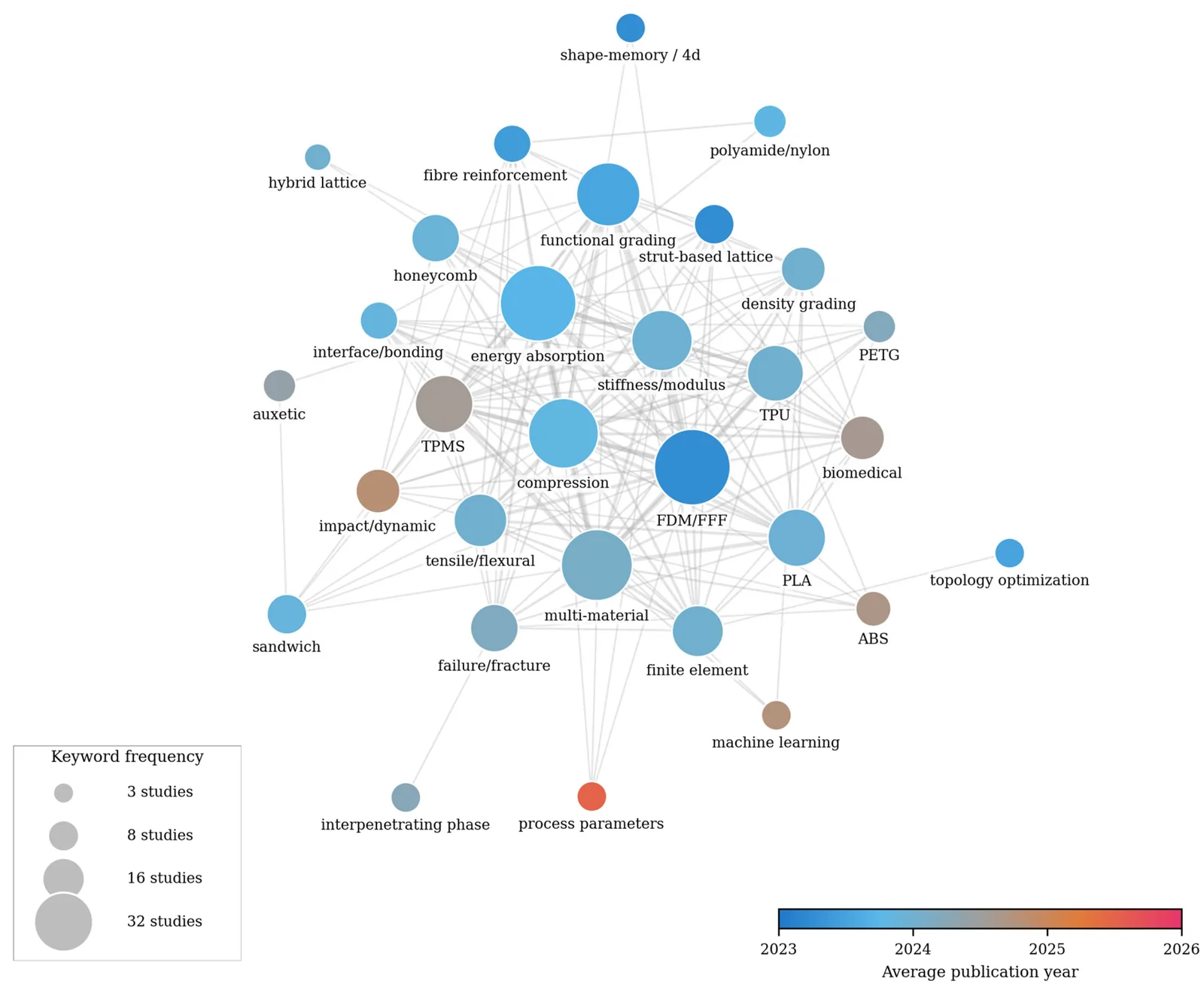
Co-occurrence network of concepts in the 65 core studies. Concepts come from a 33-term thesaurus matched against titles, abstracts and author keywords (Supplementary Material S4). Node size shows the number of studies containing a concept, link width shows the number of studies containing both concepts, and node color shows the meaning of publication year. Nodes require at least three studies and link at least three shared studies. The strongest links join energy absorption with compression testing and multi-material design, and FDM/FFF with compression testing, stiffness and functional grading, while process parameters, impact loading and machine learning have the most recent mean publication years.

Figure 3 and Figure 4 were produced with a Python script (Python 3.11, matplotlib 3.10, NetworkX 3.6; Supplementary Material S4); no dedicated bibliometric software was used. Figure 3 shows the number of publications per year for the 126 references. Figure 4 is a concept co-occurrence network of the 65 core studies. Author keywords were available for only 36 of them, so keywords were cleaned by mapping synonyms, abbreviations and spelling variants onto a 33-concept thesaurus (S4), matched case-insensitively against titles, abstracts and keywords. Each study was counted once per concept (full counting). Concepts found in at least three studies became nodes, and concept pairs co-occurring in at least three studies were linked, giving 30 nodes and 164 links; link weight is the number of shared studies.

### 2.4. Departures from PRISMA 2020

The workflow departs from PRISMA 2020 in that Stage 1 screening was automated, duplicates were removed after screening, the Stage 3 ranking used an unrecorded AI-assisted rule, 15 records were added manually, full-text exclusion reasons were assigned retrospectively, Stage 1 scored truncated titles until the error was corrected in the corrective screening, and the classification of the references was made after screening. The searches are not exhaustive (Section 2.1), and the decisions of the corrective screening are listed in Supplementary Table S1.

## 3. Fundamentals of Multi-Material AM of Gradient Polymer Lattices

To create functionally graded polymer lattices, it is necessary to develop manufacturing processes that can precisely vary the spatial distribution of material components. From its origins as a single-material prototyping technology, FDM has evolved into an emerging area of AM for multi-material parts, enabling highly complex gradient architectural forms of lattice structures. Although the use of AM technologies offers potential benefits over traditional manufacturing techniques, such as reduced cost and increased complexity, the transition from single- to multi-component production poses several new challenges, including hardware design, thermodynamic compatibility, and the structural integrity of the interface between the deposited materials. Therefore, this section will review in detail the fundamental basis of the multi-component MM-FDM system and the physical and chemical process variables that control the creation of heterogeneous lattice structures.

### 3.1. System Architecture for Multiple Materials in MM-FDM

The ability to create functionally graded lattices depends on the design of the extrusion apparatus that controls the formation of the material gradient. There are two main types of extruder designs used today. The first type uses a single nozzle with either a passive mixing chamber or coextrusion capability. The second type uses multiple nozzles. When two nozzles are used, the system is referred to as a dual-nozzle system. Dual-nozzle systems use two extruders and two nozzles to produce parts with two different polymers in parallel or in series. Functionally graded parts are created when one layer contains a specified number of unique units of lattice structure and/or when each layer contains a combination of various materials (i.e., a stiff poly(lactic acid) shell and a flexible polyurethane core). A dual-nozzle system allows for decoupling of process parameters for different materials due to differences in melting point, but it also requires periodic recalibration and is susceptible to leakage from the unused nozzle. When a nozzle is not actively engaged, it may leak residual molten plastic into adjacent areas. This may block the small pores of lattice structures. A large distance also exists between nozzles, which limits build space and complicates path planning to avoid collisions between a non-operational nozzle and the thin lattice bars formed during printing.

To alleviate some of the limitations inherent to fixed dual-nozzle arrangements, multiple designs have been proposed, including tool-changing systems and independent dual extrusion (IDEX) mechanisms. Both types of systems permit the non-functional print head to be parked away from the area being built upon, thereby reducing the mass of the print head during movement and reducing stringing artifacts observed throughout the lattice mesh [27]. Tool changer designs also permit the inclusion of multiple diameter nozzles within a single build, allowing for a multi-scale approach to fabrication. For instance, fine-diameter nozzles can be used to create detailed lattice bar geometries, while larger-diameter nozzles can be used to create coarse scale boundaries and supports for lattices [28]. This design protects the polymer from thermal degradation in the non-functioning nozzle by allowing cooling or purging before resuming use.

**Figure 5:**
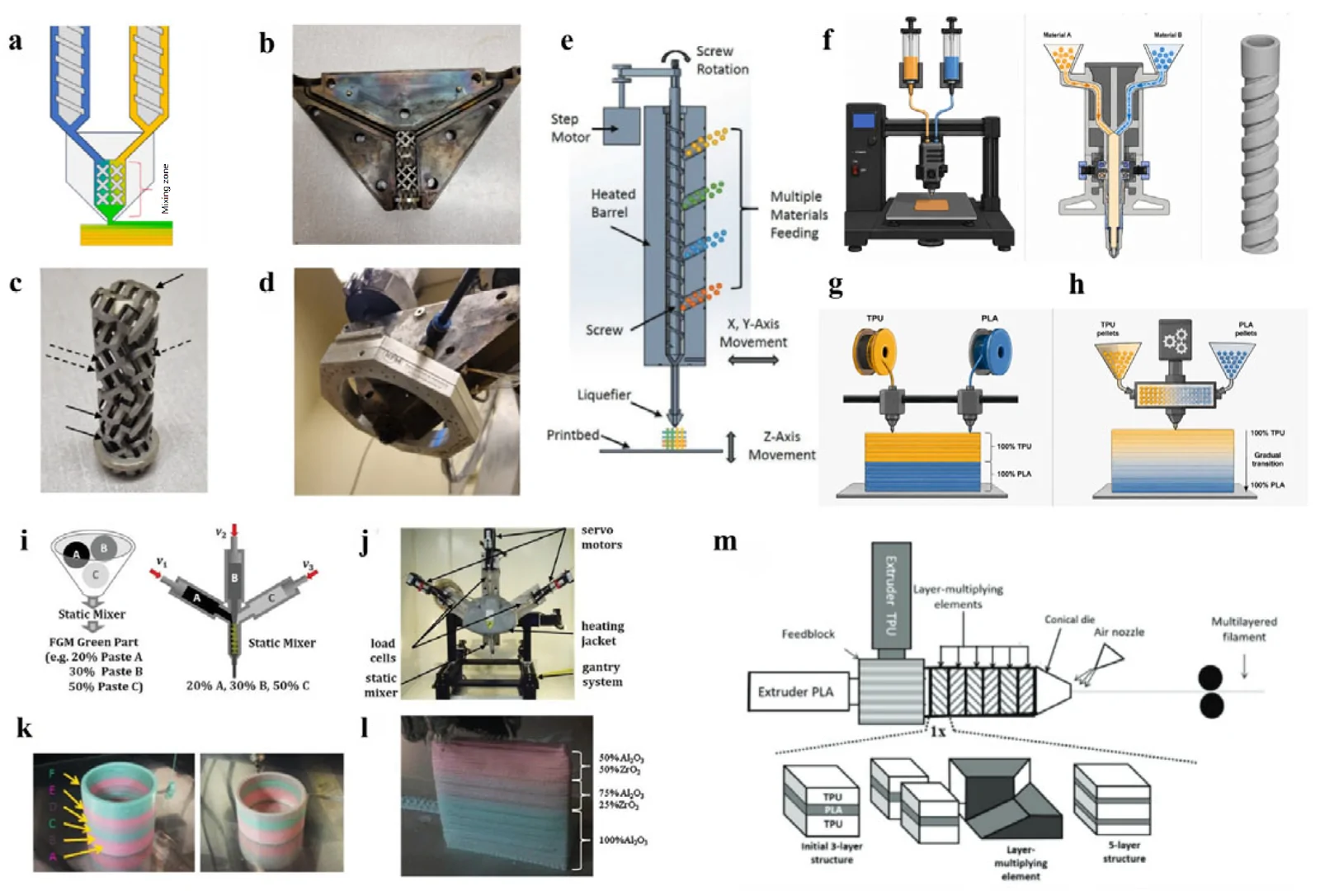
Functionally graded printing methods employing multi-material architecture and mixing materials. (**a**–**d**) Concepts for multi-material nozzles and multi-material lattices, as well as various extruder designs [23]. (**e**,**f**) Screw-based and schematic representation of multi-nozzle extrusion systems for depositing material either continuously or discretely, redrawn from Refs. [18,30]; the mixing-extruder layout in Ref. [30] comes from a ceramic system and is shown for its hardware configuration only. (**g**,**h**) Conceptual representation of conventional polymer filament extrusion utilizing separate filaments to form a layer having two distinct polymers at an interface vs. pellet-based polymer extrusion allowing for FGMs, reproduced from Ref. [11]. (**i**–**l**) Systems employing static mixers, multi-inlet printing systems, and printed components exhibiting a composition grade throughout the printed volume [18]. (**m**) Layer Multiplying Extrusion method for producing layered filaments with control over the placement and quantity of each material within each layer [31].

In addition to preventing thermal degradation of polymers in idle nozzles, IDEX mechanisms provide several advantages over traditional dual-nozzle mechanisms. They can create continuous functional gradations that are not possible when transitioning between discrete nozzle configurations. Hybrid extrusion and single nozzle mixing technologies are necessary to achieve continuous functional grading. Mixing within a chamber attached to a single nozzle or co-extruding two or more feedstocks as a blended stream prior to deposition [20] permits dynamic alteration of blending ratios of feedstock (i.e., variations in the ratio of rigid to elastic pellets), which produces continuous stiffness gradations within single lattice bars [11,23]. More recently, advances in core–shell co-extrusion nozzles have expanded design options for lattice structures by embedding reinforcing fibers or secondary polymers within a primary matrix, which increases the buckling resistance of slender lattice members while maintaining surface quality [29]. In addition to improving blend uniformity in graded lattices, recent studies have demonstrated that pellet-based fused granular fabrication (FGF) yields greater blend homogeneity than filament-based methods [11]. Figure 5 summarizes representative multi-material printing architectures and mixing strategies discussed above.

### 3.2. Compatibility for Polymer Lattice Structures

Thermal and rheological compatibilities are key factors governing the successful fabrication of multi-material lattices using polymers. Large differences in the coefficients of thermal expansion (CTEs) of the individual components can create residual stresses and/or lattice distortion during cooling, as well as delamination between the graded layers [9,20]. Similarly, processing high-temperature thermoplastics such as PEI with lower-temperature polymers will create large thermal gradients within the lattice structure. Such thermal gradients can cause the lattice geometry to become distorted during cooling [32]. Viscosity mismatches have been identified as another major barrier in both co-extrusion and mixing processes. When there are large viscosity differences between two melts, i.e., when combining a stiff thermoplastic with an elastic polymer (as observed when combining many rigid thermoplastics with many elastomers), the less viscous melt stream will encapsulate the more viscous melt stream or generate flow instabilities, causing variations in strut diameters and thus variations in mechanical properties [11].

**Figure 6:**
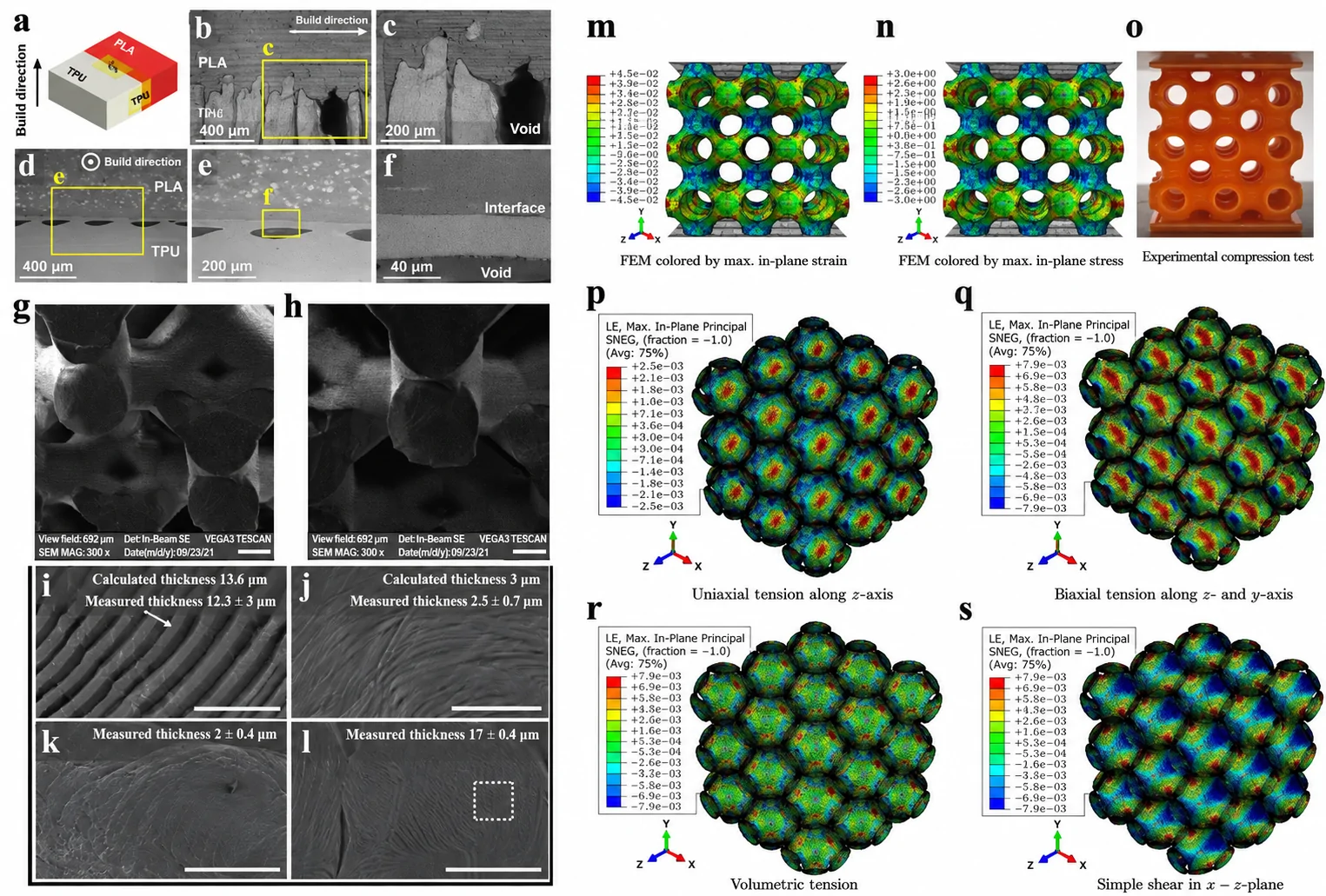
Interfacial characterization, defect formation, and numerical analysis of multi-material FDM lattice structures. (**a**–**f**) Microscopic optical evaluation of interfaces formed between PLA-TPU layers built in a three-dimensional manner to assess build directionality, interfacial morphology, and surface voids on the side and top surfaces of the interface; the arrow gives the build direction, and each yellow box marks the region enlarged in the panel named inside it [6]. (**g**,**h**) Different morphologies of fault zones under SEM, shown for comparison from a stereolithography lattice study [34]; not representative of FDM-specific interfacial defect morphology. (**i**–**l**) Microscopic and scanning electron microscopy dual-material PLA/TPU structures demonstrating printing defects, cross-sectional images of multilayer filaments before and post-extrusion, and complex three-dimensional prints; the dashed box in (l) is retained from the source figure [31]. (**m**–**s**) Numerical simulations and in-plane strain field analysis were performed on graded lattice structures subjected to mechanical loads, shown for comparison from a stereolithography (MSLA) study [15]; the color scales give the maximum in-plane principal strain (**m**,**p**–**s**) or stress (**n**).

The mechanical performance of a multi-material lattice structure is determined by the degree of adhesion at the interface between the incompatible polymeric materials. In FDM, adhesion is controlled by the amount of heat generated during processing, which allows for polymer chains to diffuse and intertwine (reptate) across the interface [20]. For compatible systems, such as different grades of TPU, strong adhesion can be achieved due to interdiffusion [23]. For thermodynamically immiscible pairs, which are commonly employed to achieve maximum property gradients, e.g., PLA/TPU or PLA/PETG, very little adhesion occurs at best due to mechanical interlocking instead of chemically bonded connections [11,23]. Bacciaglia et al. [33] quantified this for PLA/PETG in FFF, reporting an approximately 20% lower ultimate tensile strength and a 63% lower support-removal force at the dissimilar interface, with the flushing (purge) volume between materials as the controlling process parameter. Their aim was easy support removal, the opposite of the load-bearing interfaces considered here, but the results reflect the same limited interdiffusion between the two polymers. This weak interface becomes the critical failure point in lattice structures subjected to complex stress states, including shear and bending [11,12]. To address this, functionally graded composition transition zones have been shown to significantly increase interfacial strength by reducing local stresses and enabling partial molecular miscibility between adjacent layers [11,23]. Figure 6 illustrates representative interfacial characterization and numerical analyses of multi-material FDM lattice interfaces.

### 3.3. Manufacturing Challenges Specific to Multi-Material FDM

Printing lattice structures using multi-material FDM presents unique challenges compared to printing solid parts. The inherent discontinuity in lattice cross-sectional areas exacerbates interlayer bonding and reliability issues at material interfaces. As a result of the lack of substantial contact between layers in strut-based lattices, the time and surface area available for diffusion bonding are minimized [12], which increases the likelihood of delamination at multi-material interfaces within struts under load [20]. The sequencing of deposited materials directly impacts bond strength. Depositing a higher-temperature material on top of a lower-temperature substrate can facilitate remelting and diffusion, whereas depositing a lower-temperature material on top of a higher-temperature substrate results in cold bonds [35].

Additional obstacles presented include contamination between materials and interactions between print paths. Due to the nature of printing lattice structures, the print head is forced to make traveling moves between disconnected struts. Therefore, it is essential to optimize retraction settings to minimize oozing (stringing) of secondary materials into the open pore space of the lattice structure. Oozed material can alter both the relative density and the permeability characteristics of the lattice structure [17,36]. Tool path generation for multi-material lattices also requires sophisticated methodologies to accommodate transitions between materials. Crossing strategies increase interlocking between materials but may adversely affect geometric accuracy, whereas bypassing strategies preserve geometric fidelity but provide substantially weaker and more discrete reinforcements [12].

Achieving accurate geometric representation and maintaining dimensional integrity is particularly challenging in low-density lattice structures. Strut dimensions in these structures approach the diameter of the print nozzle, leading to sensitivity to flow-rate changes and dynamic lag between acceleration and deceleration events [21,37]. Transient flow instability induced by switching nozzles or materials during multi-material printing can cause under-extrusion or blobbing at material interfaces [23]. Anisotropic behavior also arises from layer-by-layer deposition methods. Mechanical responses exhibited by graded lattice structures depend upon the orientation of the lattice with respect to loading directions [2,12]. These geometric discrepancies, combined with multi-material interfaces, necessitate optimized process development and extensive characterization efforts to develop predictive capability for numerical models of FGL structures [21]. The broader design for additive manufacturing (DfAM) literature likewise identifies the characterization of manufacturing constraints and the lack of lattice-specific process standardization as open problems across AM processes [38,39].

Table 2 brings together the process parameters governing MM-FDM of graded lattices, the mechanism by which each acts, and the evidence available from the core studies. The parameters form a chain. Thermal settings (nozzle, bed and chamber temperature, and the temperature history at the interface) control melt viscosity and interdiffusion; flow settings (print speed, extrusion rate, material switching and purging) control how much material is deposited and how cleanly one material replaces another; and geometric settings (layer height, nozzle diameter and raster or build orientation) control bead size, strut fidelity and anisotropy. Together they set interfacial bonding, local density and, ultimately, the mechanical response of the lattice. Two patterns emerge. The parameters with direct evidence in the core studies are mainly those that change the deposited geometry or density, such as print speed, flow rate, layer height and nozzle diameter [19,40,41]. The parameters governing bonding at the dissimilar interface are the least studied: bed temperature is reported only as a fixed setting, chamber temperature is not reported, and no core study varied the purge volume or the deposition sequence. This reporting gap limits comparison of interfacial performance across studies (Section 10.2).

## 4. Functional Grading Strategies in Multi-Material FDM Lattice Structures

### 4.1. Material-Based Grading Strategies

Material-based grading methods depend on hardware classes introduced in Section 3. As noted previously, the ability to create different grades depends on whether the system includes dual nozzles or tool-changing to grade discretely between polymer-based zones, or single-nozzle active-mix extruders, which allow for continuous gradation of polymer content. Hence, the discussions here distinguish between these two regimes wherever the cited studies make the distinction explicit. Material-based functional grading in fused deposition modeling (FDM) has evolved beyond simple geometric tessellations to include chemically varied compositions and graded mechanical impedance at each location within a lattice structure [18,46]. Material-based functional grading primarily employs discrete zoning techniques, in which different regions of a lattice are supplied with distinct polymeric materials to maximize load-bearing and energy-absorbing capacities [18,46]. The dominant material pairing stiff thermoplastics (PLA, ABS) with hyperelastic TPU mimics the cortical–trabecular architecture of bone, integrating a stiff outer shell with a compliant core to simultaneously distribute stiffness and enhance toughness [6,16]. Studies identified in the corrective screening apply the same principle, with mixed results. Placing TPU in the stress-concentration regions of PLA porous structures printed with a dual-extruder FDM machine raised their specific yield strength and resilience [47,48]. Dual-material PLA/TPU TPMS lattices showed higher load-bearing capacity and more stable performance than their single-material constituents [49], PLA/TPU bio-inspired lattices had a steadier plateau and higher energy-absorption efficiency than the PLA-only lattice, although their SEA was lower [50], and TPU/PETG biomimetic honeycombs collapsed layer by layer under impact instead of fracturing globally, although under quasi-static compression the PETG-only honeycombs absorbed more energy [51]. Layered PLA–PETG TPMS lattices [52], topology-optimized PLA/TPU cells for non-pneumatic tires [53] and FDM PLA auxetic lattices with cast resin [54] extend the approach to other material pairings. A graded lattice combining a PLA gyroid region with a wood–PLA Schwarz-primitive region had higher compressive strength, stiffness and specific energy absorption than the uniform PLA gyroid and wood-PLA Schwarz-primitive lattices [55], and infiltrating FDM PETG lattices with epoxy raised the compressive strength of the two lattice types from 27.65 and 19.60 MPa to 36.73 and 21.87 MPa [56].

Material differences in FDM-lattice structures significantly affect their functions. For example, core–shell strut designs with a rigid PLA shell around a soft TPU core leverage the confinement effect. The rigid PLA shell prevents the strut from buckling under load, while the soft TPU core absorbs energy, thereby providing significantly greater specific energy absorption (SEA) than would be possible with a solid strut made of a single polymer [6,12]. Discrete zoning also enables designers to program how structures deform. By inserting “hard” phases into “soft” matrices, stress paths can be deflected, modifying overall failure mechanisms from catastrophic shear failures to progressive layer-by-layer failures [46]. Nevertheless, material-based grading is fundamentally constrained by interfacial compatibility: thermodynamic incompatibility between dissimilar polymers produces weak diffusion bonds that act as stress concentrators and as sites of premature failure, as characterized in Section 6.4 [9].

### 4.2. Geometry-Induced Grading in Multi-Material Lattices

Geometric grading represents another form of gradient modification in addition to material grading. Geometric grading modifies structural responses by altering relative density (ρ) and unit-cell topology [2,57], decoupling stiffness from mass and permitting precise manipulation of mechanical performance in multi-material lattices [2,25]. There are two primary means to achieve geometric grading: adjusting strut thickness (thickness grading) and/or unit cell size (size grading) [2,58]. Thickness grading is widely used to reduce interfacial stress concentrations caused by abrupt changes in local stiffness [42,46]. Zhang et al. [25] supplemented this perspective with a thorough literature review of variable-density lattice approaches for AM, detailing comparisons among thickness-grading, size-grading, and topology-transition approaches across various unit cells. Experimental studies demonstrate that lattices with thickness-gradient behavior exhibit a unique layer-wise collapse mode, enhancing energy-absorption capabilities compared to the global failure behavior of lattices with uniform densities [2,57].

**Figure 7:**
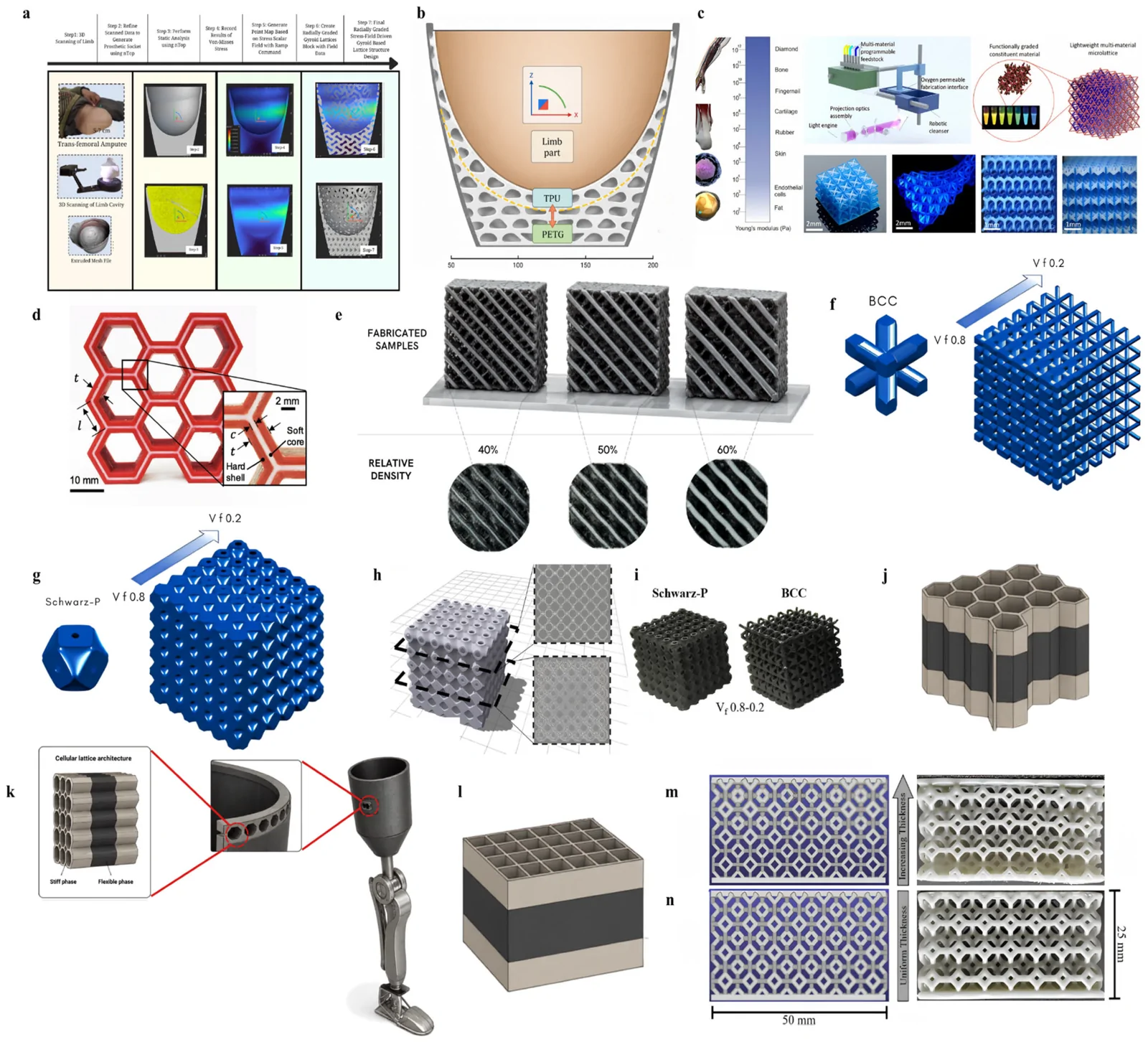
Representative strategies for functional grading and architected-lattice fabrication in multi-material FDM systems. (**a**,**b**) Stress-field-driven functionally graded gyroid generation and sectional view of TPS multi-material limb design, reproduced from Ref. [73]. (**c**) Multi-material microscale lattice fabrication using PμSL showing the printing setup, bimaterial microlattices, and isotropic polymer lattice architectures [18,74], an adjacent non-FDM (vat photopolymerization) process shown for comparison. (**d**) An FFF-printed honeycomb lattice [6]. (**e**) Schematic and fabricated multi-material diamond lattice structures illustrating the influence of geometric design and relative density on metamaterial properties, reproduced from Ref. [16]. (**f**–**i**) Continuously graded BCC and Schwarz-P lattices showing build-direction-dependent grading parameters and corresponding printed SFR specimens [57]. (**j**–**l**) Representative prosthetic leg application and design configurations of square and hexagonal multi-material honeycombs with ABS and TPU distributions, redrawn from Ref. [36]. (**m**,**n**) Rendered and fabricated Kelvin lattice structures showing vertically graded and non-graded SIL30 configurations; SIL30 is a photopolymer printed by vat photopolymerization and combined with extruded TPU [46].

Conversely, size grading adjusts unit-cell dimensions while maintaining a constant strut thickness. Size grading is especially advantageous in TPMS architectures, where continuous grading preserves smooth surfaces [14,58] (see also [59], an adjacent stereolithography study); however, thickness grading was found to yield higher SEA values than size grading in strut-based lattices due to improved load transfer [12,44]. The relationship between the geometric distribution of struts and lattice topology is critical; stretching-dominated topologies (such as octets) exhibit high stiffness but brittle failure characteristics, whereas bending-dominated topologies (such as diamonds) provide relatively high, stable plateau stresses [2,45,60]. Gradients from stretching-dominated to bending-dominated topologies permit the optimization of both initial impact resistance and subsequent energy dissipation [2,45,61]. FDM studies added in the corrective screening confirm layer-wise collapse but qualify its energy benefit. Stepwise wall-thickness grading increased the large-strain load-bearing capacity of TPU honeycombs [62] and the specific energy absorption of PLA honeycombs [63], while in PLA and fiber-filled PLA cellular structures, densifying the cells at the center raised SEA only slightly (2.95 vs. 2.81 kJ/kg) and densifying them at the faces lowered it [64]. Grading the unit-cell angle of PLA auxetic double-arrowhead lattices raised specific energy absorption by up to 173% over the uniform design with the smallest angle but by only 1.4% over the best uniform design [65] and graded auxetic sandwich cores absorbed a larger share of the impact energy than uniform auxetic cores, although a uniform honeycomb core had the highest SEA [66]. Density and unit-cell-size grading were studied in short-carbon-fiber nylon lattices [67], layer-wise changes of unit-cell type in PLA [68] and carbon-fiber nylon lattices [69] (where, at equal relative density, the layered lattices did not absorb more energy than the uniform cubic lattice), and load-path-driven porosity grading [70] and multiscale topology optimization [71] in FDM-printed PLA-based lattices. A beam filled with graded, topology-optimized lattice cells and produced by material extrusion reached 1.6–1.9 times the effective stiffness and twice the ultimate load of a conventionally designed beam [72]. Representative geometry-induced and architected-lattice strategies discussed are illustrated in Figure 7.

### 4.3. Combined Material–Geometry Grading Approaches

**Figure 8:**
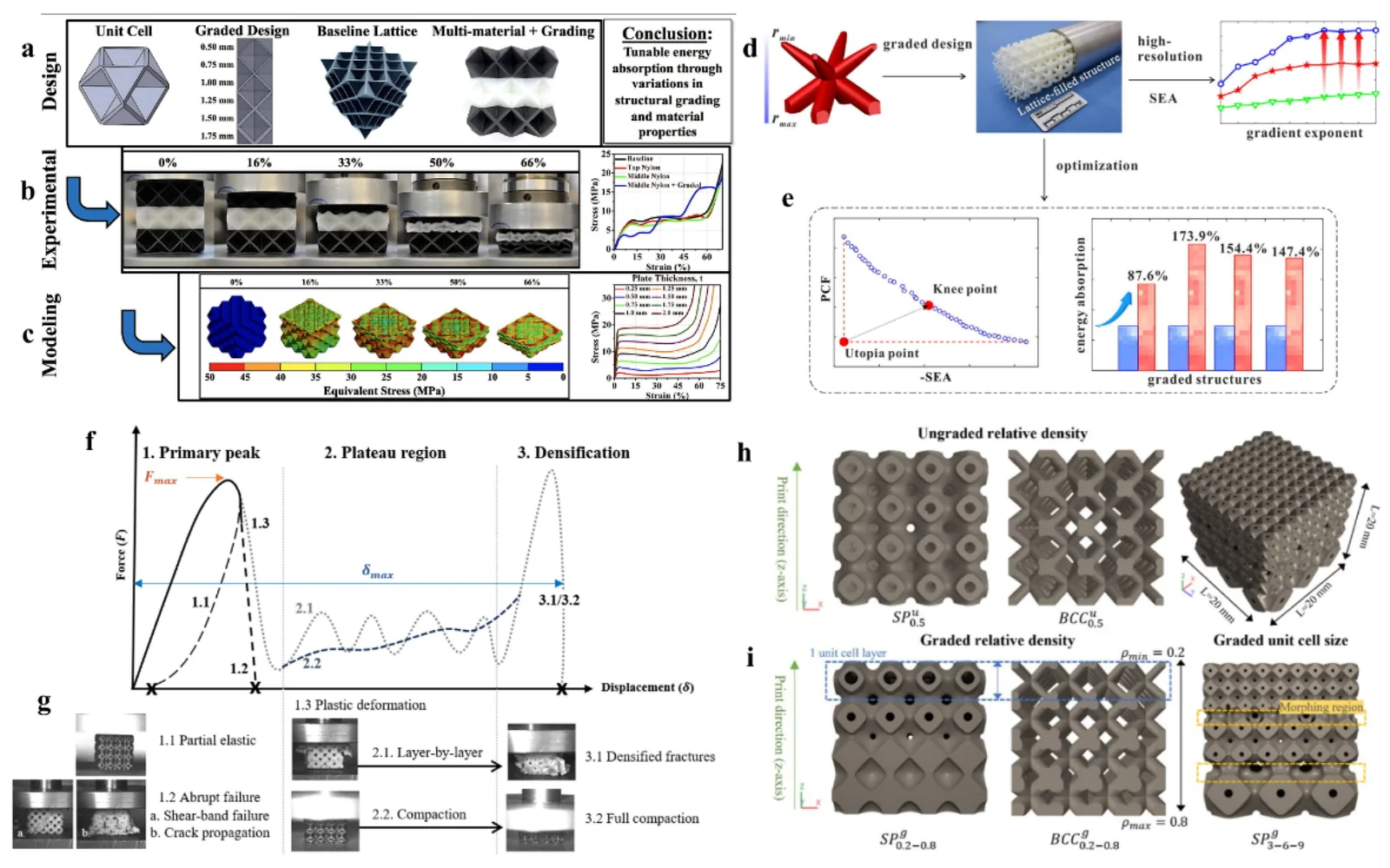
Combining material grading and geometry grading approaches and their effect on the mechanical properties of multi-material lattice structures. (**a**–**c**) Designing and experimentally characterizing and numerically modeling structurally graded octet lattices with varying relative densities and material distributions [42]. (**d**,**e**) Optimization-driven grading strategies designed to improve SEA and collapse behavior, shown for comparison from a stereolithography study [59]. (**f**–**i**) Representative lattice dimensions, examples of graded specimens, and force–displacement response curves with deformation modes [44].

Combining material and geometry gradients yields a synergistic enhancement in mechanical properties that far exceeds that achievable with single-parameter grading [42]; a similar synergy was reported in an adjacent stereolithography study [59]. Combined methodologies utilize independent control over material stiffness and structural density to satisfy conflicting design objectives, such as maximizing energy absorption while minimizing the maximum transmitted forces [42,46]. An exemplary application of the combined methodology is vertical stackings of unit cells with varying plate thicknesses and material compositions. Studies utilizing lattices exhibiting both structural grading (strut thickness variegation) and material grading (alternate layers of reinforced vs. unreinforced polymer) demonstrated the capability to manipulate the order-of-collapse of individual layers [42]. In these “Dual-Graded” structures, deformation begins in the least stiff geometrical/material layer, lengthening the plateau area of the stress–strain curves, resulting in enhanced total energy absorption [42]. This can be described in Zhao et al.’s research [75], who developed an optimum design process for multi-material heterogeneous lattice energy absorbing structures. Figure 8 illustrates combined material–geometry grading strategies and their effects on mechanical performance.

**Figure 9:**
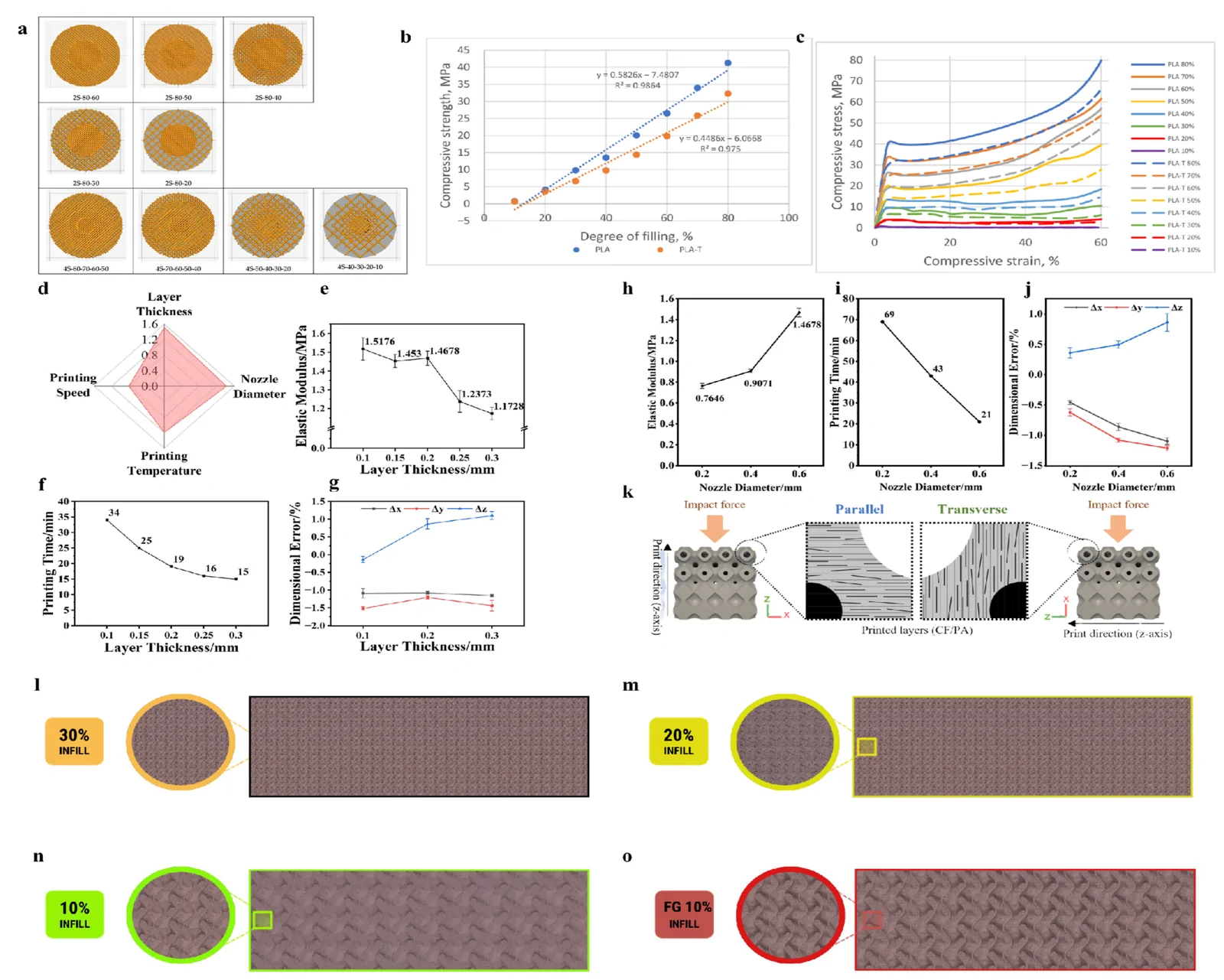
Process-induced grading strategies and their influence on the mechanical behavior of multi-material FDM lattice structures. (**a**–**c**) Visualization and mechanical characterization of multi-zone graded models showing compression behavior and elastic modulus variation with infill density in PLA and PLA–T structures [43]. (**d**–**g**) Range analysis of the process parameters, the effect of layer thickness on compressive elastic modulus, print time, and dimensional error, respectively, [41]. (**h**–**j**) Nozzle diameter effect on printing performance in terms of compressive elastic modulus, print time, and dimensional error [41]. (**k**) Printing direction based on parallel and transverse directions [44]. (**l**–**o**) Toolpath preview of the four printed specimens: from top to bottom, 30%, 20%, and 10% uniform-density, and FG10% Gyroid lattices, redrawn from Ref. [19].

The use of radial hybridization in combination with functional gradation has also shown promise. Hybrid cylindrical lattices constructed using distinctly different unit cell types arranged radially within a single structure (e.g., octahedral, FCC, BCC), but differing in their relative densities, have been found to offer improved compressive modulus and ultimate tensile strengths when compared to equivalent uniform hybrid structures [37,61]. These hybrid structures are formed by stiffer outer layers that encase softer core areas, thereby reducing the likelihood of global buckling in the structure and promoting localized plastic flow prior to failure, ultimately increasing the SEA [61]. Combining additive manufacture of multiple materials with spatially graded geometries helps mitigate the effects of additive manufacture induced anisotropy, resulting in structures having more isotropically responsive properties [37,44,45]. However, successful realization of FGLs requires advances in predictive computational models capable of accurately predicting the effects of interaction between material interfaces and spatially varying geometries on both residual stresses and Poisson’s ratio mismatches [15,42,58,76]. Process-induced grading strategies and their effects on mechanical performance are shown in Figure 9.

### 4.4. FDM Process Induced Grading

Functional gradation can also be achieved in FDM through “process-driven” methods, in which deposition parameters are modulated to induce density variations without requiring modification of the CAD geometry [19]. One recent example of this type of process modulation is dynamic variation in printhead velocities during deposition. Varying deposition rates will result in varying densities; therefore, lower velocities will yield higher density/stronger struts, and conversely, higher velocities will yield lower density/porous regions [19]. This type of processing enables the generation of functionally graded structures from unmodified STL files, thereby streamlining the process [19].

Layer-by-layer material switching using multi-nozzle systems enables discrete zoning along the build axis, allowing for the creation of composite sandwich lattice structures consisting of a stiff skin and core combination [35,36]. In its most basic form, varying the infill percentage in FDM-printed PLA samples results in detectable stiffness gradations without modifying the tool path geometry used to create them [43], thus verifying the process grading concept. Interfacial debonding issues are addressed using print path strategies, such as alternating infills and topological interlocking weaves, to mechanically interlock dissimilar materials at the interface, thereby improving resistance to delamination [77,78,79]. An additional benefit of process-induced anisotropy is achieved through changing the orientation of rasters or build axes and subsequently aligning fibers with principal stress directions to maximize performance [44,45]; orientation-dependent strut properties have also been modeled in an adjacent vat-photopolymerization study [80]. Together, these process-induced approaches provide a wide range of options for creating complex functionally graded polymer lattices [81]. In an orthogonal approach, Zhao et al. [40] employed a complementary approach in which they integrated thermally expandable microspheres into TPU extrusions to generate multi-level microcellular gradients, extending the scope of process-driven gradient formation beyond that based on speed modulation. Table 3 summarizes and compares all four functional grading strategies discussed above, along with their typical implementations, reported performance benefits, and key limitations.

## 5. Lattice Architectures Used in Multi-Material FDM

The topological design of component unit cells directly influences the effectiveness of functionally graded components. With respect to MM-FDM, the selection of lattice topology depends on two primary factors: mechanical efficiency, defined as stiffness-to-weight and strength-to-weight ratios, and manufacturing limitations, including self-supportability, continuous interfaces, and nozzle accessibility [21,60,84]. Lattice architectures are generally classified into three broad categories, including strut-based (truss) systems, surface-based (triply periodic minimal surface, TPMS) systems, and hybrid topologies. Each category interacts differently with multi-material extrusion capabilities, thereby presenting unique trade-offs among stiffness, toughness, and recoverability [2,85].

### 5.1. Strut-Based Lattice Architectures

Lattices composed of strut- or beam-based designs that create interconnected periodic networks are among the most basic forms of cellular solids manufactured using AM techniques. There are two primary ways in which lattice-based architectures can be categorized according to the Maxwell stability criterion: bending-dominated (e.g., BCC, honeycomb) and stretching-dominated (e.g., octet-truss, FCC) [85,86]. Mocerino et al. [87], produced an exhaustive literature survey of FDM-fabricated polymeric auxetic structures. This review was developed around a framework for categorizing and describing re-entrant, chiral, and rotating unit topologies related to graded strut design. Joseph et al. [88] conducted a systematic review of the processing conditions used to fabricate auxetics using AM. In addition, they noted the limitations on the geometric accuracy of re-entrant unit cells imposed by FDM’s resolution limits. The majority of stretch-dominated lattices exhibit higher specific stiffness and ultimate strength than other lattice architectures. However, they also tend to experience catastrophic post-yield softening due to instability in individual struts under compressive loading [86,89]. On the other hand, bending-dominated lattices tend to exhibit longer stress plateaus and progressive densification as stiffness decreases, and therefore may be better suited for use in energy-absorbing applications [45,90].

Strut-based lattice structures enable the creation of discrete functional gradients in MM-FDM using selectively deposited polymers with different physical properties. A large body of research has focused on bio-inspired honeycombs and re-entrant auxetic structures to study how stiff cores contribute to structural integrity, while soft cores increase the ability to absorb energy [6,36,91]. As examples of multi-material hexagonal lattices fabricated using MM-FDM, Yavas et al. showed that a variety of material combinations and fabrication methods could produce energy absorption capacity values 2–3 times those that would result from single-phase constituents alone, depending on the core-to-strut thickness ratio [6]. While strut-based designs have proven to be effective in achieving graded property distributions in FDM, there are many practical challenges associated with creating strut-based lattice structures in FDM. First, all lattice nodes serve as localized sites for stress concentration; however, these stresses become even more concentrated when thermodynamically incompatible polymers intersect at these lattice nodes [5,6,35]. Low-relative-density lattice-based strut configurations frequently present difficulties in maintaining accurate geometry and minimizing strut wavy deformations during extrusion, as the process of accurately determining the degree of fidelity associated with producing very thin diameter struts (less than approximately 5mm in cell size) remains problematic [21].

### 5.2. Surface-Based Lattice Architectures

TPMSs have evolved into an attractive replacement for strut-based lattices in MM-FDM, as they possess inherent topological continuity, can support themselves without external support, and are characterized by a mathematically defined zero mean curvature [15,92]. Common architectures such as the Gyroid (G), Schwarz Primitive (P), and Diamond (D) partition space into continuous, non-overlapping sub-spaces, thereby allowing the production of interpenetrating phase composites [93,94]. The relationship between the geometry and mechanics of each surface type has been well characterized: Hou et al. [95] mapped the relationship between the elastic anisotropic behavior and the skeletal geometry of the Gyroid in powder-bed-fused Ti6Al4V (an adjacent process–material system, cited for comparison), providing cross-material benchmarks.

TPMS-based architectures are especially beneficial for developing multi-material gradient distributions, as the continuous nature of their interfaces eliminates sharp nodal stress concentrations that occur in trusses, thereby delaying interfacial debonding [37,96]. The mechanical responses of TPMS architecture can be controlled over a wide range. For example, Diamond tends to exhibit higher stiffness and yield strength, making it more suitable for load-bearing applications, whereas Gyroid provides a good balance between energy absorption and isotropy [37,93]. Recent studies on PLA/PETG and PLA/TPU IPCs indicate that the thermal compatibility between phases in TPMS arrangements enables in situ bond formation, thereby preventing cracking and extending the time to failure via plastic deformation [94,96]. The development of graded TPMS structures via FDM creates both computational and fabrication challenges. Developing continuous tool paths necessary to generate graded density distributions requires sophisticated slicing strategies to minimize defects near transitional zones and to ensure the precision needed to maintain dimensional fidelity across varied wall thicknesses [19,97]; comparable requirements have been reported in an adjacent laser powder-bed fusion study [98].

### 5.3. Hybrid and Topology-Transition Lattices

As both strut and surface topologies have their own limitations, researchers have recently developed “Hybrid” lattices that spatially combine multiple unit cell types to achieve synergistic mechanical behavior. In addition to Longitudinal Stacking (Multi-Layering), Radial Nesting, and Logic-Based Superimposition of Architectures, hybridization strategies in MM-FDM are realized [61,85].

**Figure 10:**
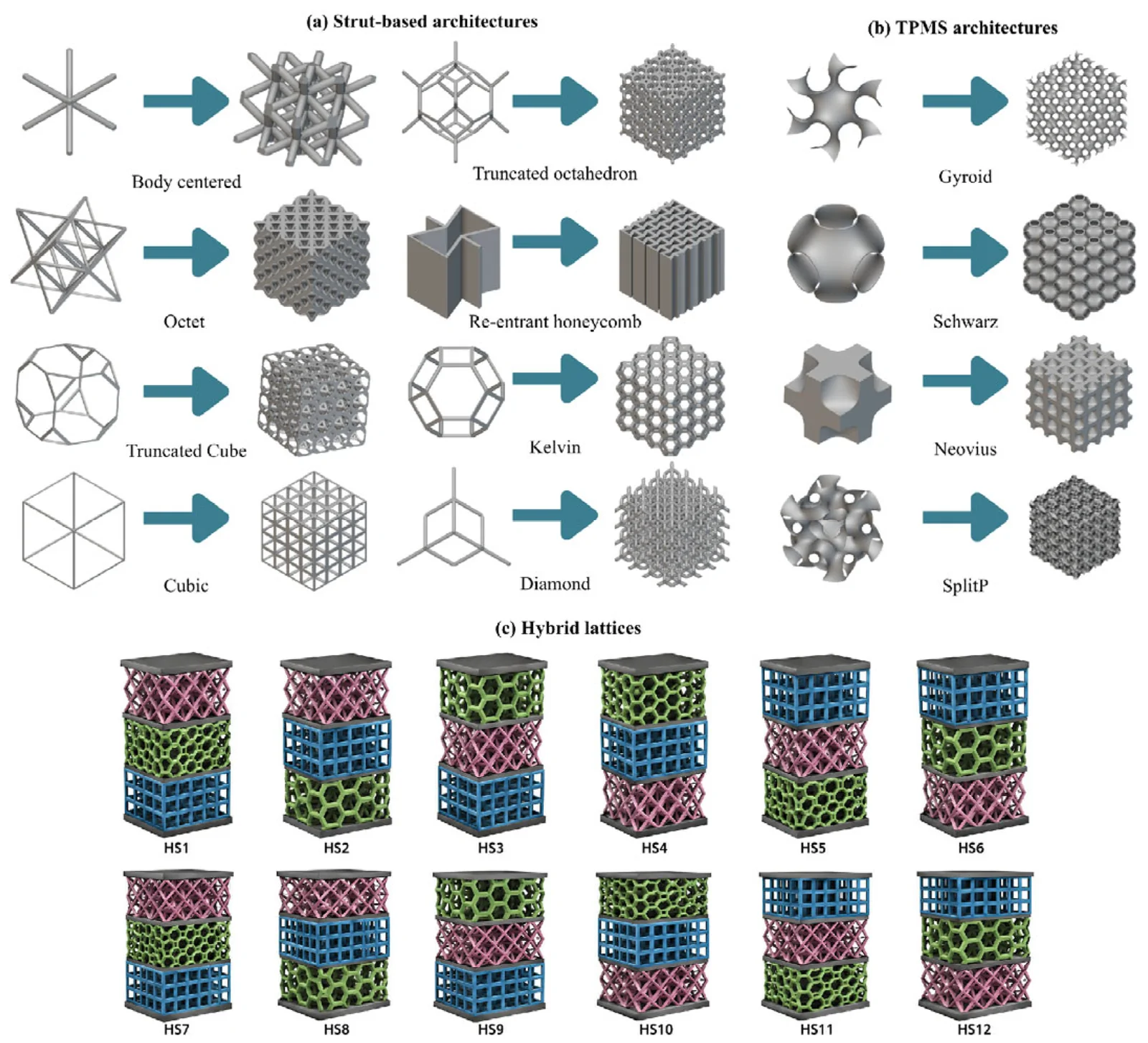
Classification of lattice architectures used in multi-material functionally graded structures. (**a**) Representative strut-based architectures, including re-entrant, kelvin, diamond, octet, and cubic lattices, redrawn from Ref. [12]. (**b**) TPMS architectures including Gyroid, Schwarz, Neovius, and SplitP geometries, redrawn from Ref. [12]. (**c**) Hybrid lattice configurations combining multiple architectures through layered topology transitions for programmable stiffness, deformation control, and enhanced energy absorption, redrawn from Ref. [85].

One of the most promising new strategies is the “Plate–TPMS” hybrid. This architecture includes embedding strut- or plate-based lattices into the void space of TPMS shells, e.g., simple cubic plates reinforced with Schwarz Primitive shells [37]. Naji et al. showed that hybridizing simple cubic (SC) plate lattices with either Diamond or Gyroid TPMS lattices could produce up to 171% increases in stiffness and 125% increases in yield strength compared to their elementary TPMS counterparts. They indicated that hybrid Plate–TPMS architectures could outperform current hybrid architectures [37]. Similar results were obtained using multilayered hybrids, including BCC (soft/bending-dominated) and Octet (stiff/stretching-dominated) lattices. Researchers have shown that multi-layered hybrids capable of programming progressive collapse modes yield SEA values substantially greater than those of homogeneous lattices [85]. Radial hybridization, which utilizes a bending-dominated core encased by a stretching-dominated shell, has been shown to effectively suppress global buckling and enforce stable layer-wise deformation [61]. The findings presented by Bastola et al. [99], an adjacent selective laser sintering study, supported these findings by demonstrating that polyamide hybrid lattices with locally reinforced struts produce superior energy absorption and stiffness due to the combined effects of topology hybridization and material reinforcement of struts at a local scale, which were confirmed both numerically and experimentally. However, these architectures utilize multi-material depositions to place high-strength materials in regions of maximum stress and low-strength materials for damping, but at the cost of requiring thorough optimization to manage the large stiffness discontinuities at topology transitions [90]. Figure 10 classifies the strut-based, TPMS, and hybrid lattice architecture discussed in this section.

### 5.4. Design Considerations for Multi-Material Lattice Realization Using AM

Realizing functionally graded polymer lattices using AM processes requires the careful consideration of DfAM constraints unique to multi-nozzle extrusion.

- Interfacial Integrity and Bonding: The interface between two dissimilar polymers is the weakest link in MM-FDM lattices. Thermodynamically incompatible polymers (e.g., PLA/PP or ABS) result in poor diffusion bonding at interfaces, leading to early delamination under loading [20,23,79]. Methods to address this issue are available, e.g., designing mechanically interlocked elements (e.g., “interlaced” or “dovetail”) along the struts of the lattice, or designing functionally graded transition zones between adjacent layers to minimize stress concentrations along the interfaces [11,35].
- Printability and Geometric Fidelity: T Resolution limits inherent to FDM nozzle geometry impose a minimum value for the diameter of lattice struts. Switching between multiple nozzles in multi-material lattices introduces defects such as stringing, oozing, and under-extrusion at lattice nodes, which are potential crack initiation sites [20,21]. Studies have shown that lattice cells smaller than a certain size exhibit significant deviation from nominal dimensions and are extremely fragile [21].
- Topology–Material Compatibility: Selection of topology must match the characteristics of the material. For example, highly stiff materials are used in stretching-dominant lattices to prevent buckling. On the other hand, hyperelastic materials are well-suited for bending-dominant lattices to absorb energy [46,85]. Since FDM is inherently anisotropic (Z-strength is less than XY-strength), primary load-carrying members should be oriented along the direction of deposition or be reinforced with continuous phases in IPC configurations [76,96].

Strut-based lattices are simpler to design, whereas surface-based TPMS and novel hybrid Plate–TPMS architectures have been designed to create greater continuity and mechanical synergy among the materials used in AM processes for multi-material FDM. It is anticipated that future developments will include the development of computational methodologies to optimize material distributions and interface designs, ensuring that the theoretical advantages of functionally grading are not lost due to defects generated during manufacturing.

## 6. Mechanical and Functional Performance

### 6.1. Compressive, Tensile, and Flexural Behavior

Multi-material polymer lattice structure behavior is determined by the interplay of the two different materials used within the structure. Typically, a compliant matrix is combined with a stiffer reinforcement. Multi-material lattices have been shown to offer a number of benefits over single-material lattices, including the ability to independently control both global stiffness and strength through spatially modulating the distribution of material [6,12]. In the compressive regime, multi-material lattices composed of a rigid thermoplastic, such as PLA or acrylonitrile butadiene styrene (ABS), and a hyperelastic TPU exhibit mixed stress–strain behavior. Initially, the behavior is linearly elastic due to the stiff phase; subsequently, there is an extended period of dense plateaus due to the soft phase [6,93].

In terms of design flexibility, FGL structures are highly beneficial for load-bearing applications. Experiments have shown that increasing the volume fraction of the rigid phase in a multi-material lattice, such as PLA, results in significant increases in elastic modulus and peak stresses, consistent with a rule-of-mixtures relationship. Conversely, the addition of a soft phase to the lattice will delay catastrophic failure [16,93]. For example, in PLA–TPU gyroid structures, an increase in the amount of PLA present in the structure leads to an increase in peak stress. Conversely, an increase in TPU content in the structure enhances recoverability and ductility [6,93]. Paz González et al. [34] provided a comparative analysis of AM process parameters using SLA-fabricated lattices (stereolithography; an adjacent process, cited for comparison). Specifically, they indicated that IsoTruss geometries with linear density gradients achieved an energy absorption of approximately 15 MJ/m^3^ at a strain of approximately 44%, a useful reference point for evaluating FDM-based FG Designs. Under flexural loading conditions, multi-material struts exhibit a transition from brittle failure to progressive yielding. It has been shown that while homogeneous rigid lattices undergo catastrophic failure once the critical stress level is reached, multi-material configurations maintain some residual load-carrying capacity throughout the compliant core, thereby extending the failure strain [6,19]. Additionally, tensile characterization studies on multi-material sandwich-structured lattices have shown that multi-material configurations can achieve higher specific tensile strength than their equivalent homogeneous counterparts by limiting localized stress concentrations through compliant interfacial regions [35,92].

### 6.2. Energy Absorption and Crashworthiness

The most compelling benefit of multi-material FDM lattices is their enhanced energy absorption, driven by a programmatic progressive-collapse mechanism. Through functional grading, designers can tailor the failure sequence progression: deformation begins in layers with the least stiffness, whether geometric or material-based, and then progresses sequentially through subsequent layers to smooth out stress–strain oscillations inherent in uniform lattices [44,58,85], thereby maximizing the area under the stress–strain curve and producing high SEA. The studies added in the corrective screening confirm the layer-wise collapse of graded lattices [62,63,65,69], but the energy gain over uniform lattices of equal mass depended on strain and was often small: graded lattices absorbed more energy only at large strains [62,67], gained 2–21% in SEA [63] or 1.4% over the best uniform design [65], or did not consistently exceed the best uniform lattice [64,66,69].

The combination of stiff and soft materials produces a confining effect that greatly enhances crashworthiness. Bio-inspired designs, such as honeycombs or gyroid lattices incorporating hard shells with soft cores, exhibit SEA values that are 2–3 times larger than those of their single-phase constituents [6,16]. The size of this gain depends on the baseline and on the loading rate. Against the soft constituent alone, gains of about 2.5–4.9 times were reported [47,50]; against the rigid constituent, gains were smaller, about 1.4–2.4 times [47,49] (in [49] at twice the relative density of the PLA lattice), and in several quasi-static tests, the rigid single-material lattice absorbed more energy than the dual-material one [50,51,52]. In PETG/TPU honeycombs, the PETG-only designs had 2.1–3.2 times the SEA of the dual-material designs in quasi-static compression, whereas the dual-material designs were 35–66% better under impact at 14.9–26.6 m/s [51]. These configurations utilize the rigid shell as an initial peak-strength component, and the soft-core region to stabilize the shell against localized buckling and dissipate energy through hyperelastic deformation [6]. Under dynamic loading regimes, strain-rate sensitivity is also considered as another important factor. Studies on FGLs subjected to drop-weight impacts indicate that graded architectures provide superior energy absorption efficiency compared with uniform configurations, especially at intermediate strain rates, by promoting sequential deformation modes that inhibit global shear failure [44,46]. Kumar et al. [100,101,102] expanded on the enhancement of EA in AM polymer composite lattices by evaluating the contributions of reinforcement phase volume fractions to plateau stresses and SEA, providing comparative data across graded configurations that are directly comparable to multi-material systems. Viscoelastic effects associated with elastomeric phases (e.g., TPU) contribute to impact mitigation by reducing peak transmitted forces and damping vibration [46]. Systematic evaluations of quasi-static SEA improvements in polymer composite lattice structures subjected to compression have demonstrated that the choice of architectural type is the primary factor governing improvements in energy absorption and that composite lattices can enhance SEAs relative to their equivalent monolithic counterparts by up to 40% [103]. However, translating these quasi-static advantages into real-world application performance will depend critically on strain rate, loading geometry, and boundary conditions, which are addressed in Section 7.

### 6.3. Deformation and Failure Mechanisms

**Figure 11:**
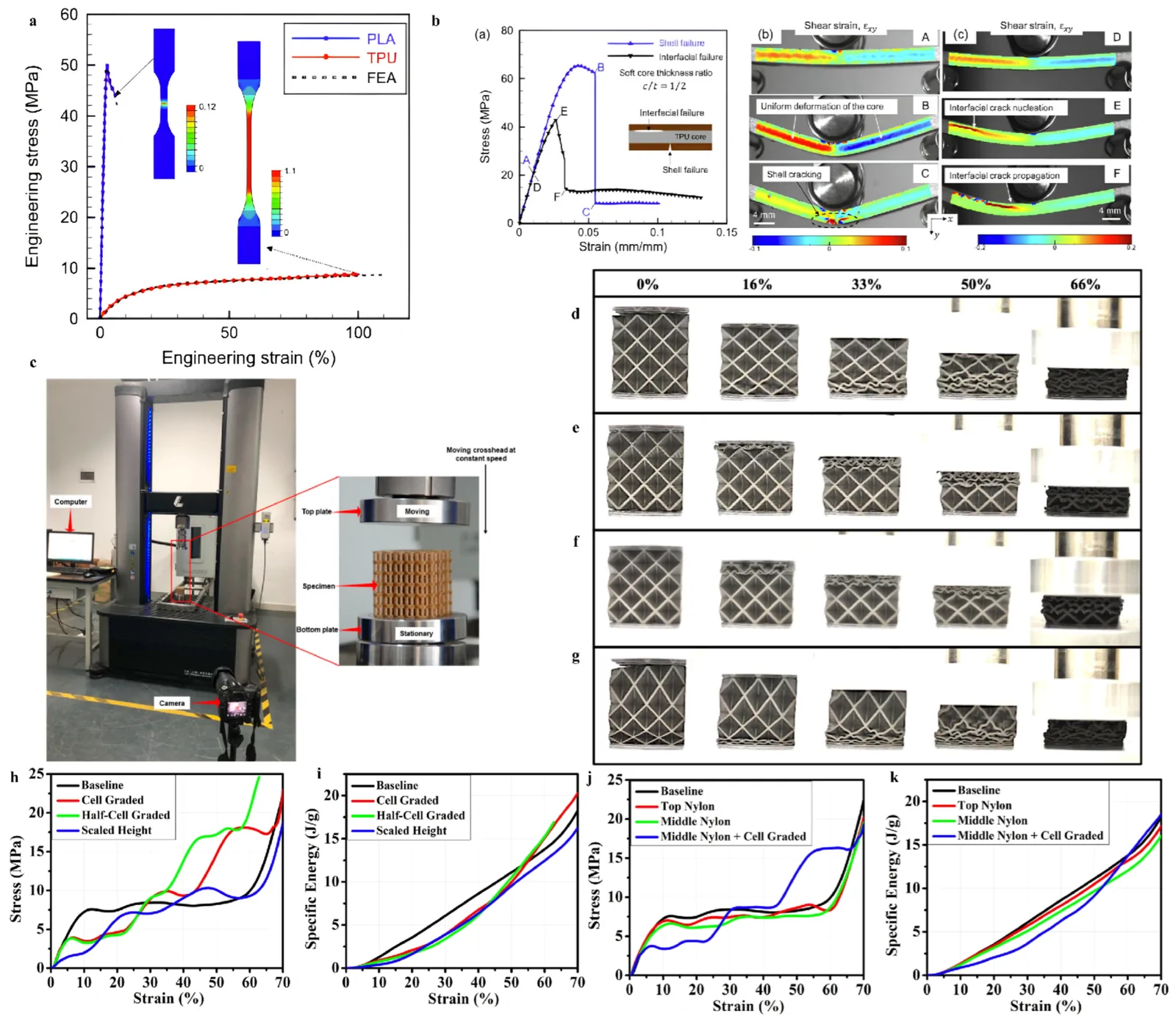
Mechanical characterization and failure behavior of multi-material graded lattice structures under compressive loading. (**a**,**b**) Experimental and finite element analysis (FEA) stress–strain response of PLA and TPU graded structures, representative deformation stages, and interfacial failure evolution during compression [6]. (**c**) Experimental compression testing setup [7]. (**d**–**g**) Quasi-static compression images captured at successive strain intervals for the (**d**) baseline, (**e**) cell-graded, (**f**) half-cell-graded, and (**g**) scaled-height octet lattices [42]. (**h**,**i**) Graded lattice structures: stress–strain curves and specific energy absorption versus strain for the baseline, cell-graded, half-cell-graded, and scaled-height lattice structures under compression [42]. (**j**,**k**) Multi-material stress–strain curves and specific energy absorption versus strain for the baseline, cell-graded, half-cell-graded, and scaled-height lattice structures [42].

Failure mechanisms in multi-material lattices, as shown in Figure 11, are complex; however, they arise primarily from interactions between the brittle fracture of the rigid polymer and the elastic buckling of the elastomer. Rigid homogeneous lattices typically fail catastrophically through diagonal shear bands or brittle fractures of individual struts [6,85]. Soft lattices fail through elastic buckling and densification prior to fracture [12]. Hybrid lattices fail through a combination of mechanisms and exhibit a unique mechanism of localized progressive buckling due to the suppression of global shear bands by the inclusion of a soft phase [6,61,85].

In hybrid lattice geometries comprising combinations of truss- and surface-based topological arrangements, the deformation sequence is governed by the relative stiffnesses of the substructures. The first region to fail within hybrid geometries will be the least stiff, typically either the bending-dominated units or the softer material layer, followed sequentially by progression into the higher stiffness, stretching-dominated regions [85]. This programmed failure provides an alternative to catastrophic loss of structural integrity; however, it also introduces additional failure modes at interfaces between different material types. Discrete zone-based approaches, such as those employing rigid and soft polymeric layers, may create stress concentrations at material interfaces, leading to premature delamination or cracking prior to full utilization of material strengths [6,35]. Consequently, the global ductility of the lattice is commonly limited by the strain level at which interfacial debonding occurs [6,11].

### 6.4. Influence of Material Interfaces on Mechanical Response

Multi-material additive-manufactured lattices are highly dependent on the quality of the interfaces formed between the various materials used to fabricate them. As a direct result of thermodynamic incompatibilities and variations in thermal expansion coefficients among materials such as PLA, ABS, and TPU, diffusion bonding between these materials is poor, leading to low-quality interfaces [11,23,35]. Using digital image correlation (DIC), experimental studies have demonstrated that the primary area of strain localization occurs at the interface between materials, leading to early failure under tensile or shear loads [6,35]. Amador et al. [104] conducted systematic investigations of multiple combinations of polymeric 3D-printing materials under various loading conditions. They developed quantitative comparisons to evaluate reductions in the effective modulus induced by weak diffusion bonding at immiscible interfaces and established metrics to assess the comparative performance of optimized interfaces.

The quality of the interface affects how effectively stresses can be transferred between the two phases. Poorly performing interfaces act as defects, significantly reducing the effective modulus and strength of the lattice relative to predicted values [6]. To address this issue, researchers have investigated several alternatives, including mechanical interlocking (i.e., jigsaw interfaces) or functionally graded transition zones. Continuously graded materials produced using active mixing nozzle technology or pellet-based extrusion exhibit improved inter-molecular diffusion and reduced interfacial stress concentrations, thereby increasing resistance to delamination and enhancing overall structural toughness [11,73]. Discretely stepped interfaces remain a significant constraint in current multi-nozzle FDM technologies [9,23].

### 6.5. Comparison with FGL Structures Produced Using Single Material

FGL structures composed entirely of a single material improve upon traditional lattice structures with respect to mass distribution [2,58]; however, single-material FGL structures are ultimately constrained by the inherent physical properties of the base material. Single-material FGL structures present designers with a fundamental trade-off: reducing density to minimize mass will simultaneously reduce stiffness and strength [4,61]. Therefore, multi-material FGL structures allow designers to separate geometric design from material properties. When a high-strength phase is integrated for load-bearing and a high-damping phase for energy dissipation, multi-material lattice structures can achieve a balance of stiffness, toughness, and recoverability that is unattainable with a monolithic system [22,46,82].

When comparing single-material and multi-material FGLs, researchers find that while single-material FGLs are advantageous for manufacturing simplicity and continuous interfaces throughout the structure, multi-material FGLs are advantageous for designing multifunctional structures capable of simultaneously absorbing impacts and recovering from them [16,46]. Unfortunately, multi-material FGL structures require more complex manufacturing processes than single-material FGL structures. Reliability issues related to interfacial defects and anisotropy in FDM-deposited structures further complicate manufacturability [9,61]. Therefore, multi-material FGL structures are optimal for applications where increases in energy absorption and specific strength outweigh the added manufacturing process complexities and costs [6,93]. Table 4 consolidates the mechanical performance and energy absorption characteristics reported across the reviewed studies.

### 6.6. Design Guidelines Emerging from the Reviewed Studies

The studies reviewed in Section 4, Section 5 and Section 6 support a small number of design guidelines, listed in Table 5. They are trends observed across studies with differing materials, topologies and test conditions (Table 4) and have not been validated as design laws. The 21 studies added in the corrective screening were checked against these guidelines: they confirm the layer-wise collapse of graded lattices and the dependence of multi-material gains on configuration, but they show that the energy gains of both strategies are smaller, and depend more on strain and loading rate, than the earlier studies suggested (Section 6.2).

Material combinations. Rigid–hyperelastic pairs (PLA, ABS or PETG with TPU) are the most frequently studied and give the largest reported energy-absorption gains, up to two- to threefold over the constituents in core–shell struts [6], although the gain depends strongly on configuration and on the baseline chosen [16,36,93]. The additional studies show that the gain also depends on the loading rate and that, compared with the rigid constituent alone, quasi-static gains were small or negative in several cases [47,49,50,51,52]. Their reliability is limited by weak interfaces between immiscible polymers, which were the failure sites in several studies [6,11]; in quasi-static compression, however, the interfaces remained intact in other studies [49,52], so their effect depends on the loading mode and print quality. Where interfacial reliability is the priority, interpenetrating TPMS arrangements, in which the interface is continuous and mechanically interlocked, are preferable [94,96].

Grading strategy by objective. For energy absorption, density or thickness grading gives progressive, layer-wise collapse [42,44,57,58,62,63]; its energy gain over uniform lattices of equal mass is strain-dependent and can be small or absent [62,65,67,69]. For stiffness at low mass, the stiffer material and higher density are placed along the load path [3,106]. For repeated impacts, TPU-rich or shape-memory phases are needed to recover the lattice [16,105]. Where design effort must be minimal, process-induced grading avoids CAD modification [19,43] at the cost of a less predictable response.

Relative density and interfaces. The reviewed studies do not support a general relative-density threshold: the ranges tested are study-specific and are rarely reported in comparable terms (Table 4). The reported practical lower bound is geometric: dimensional error grows with nozzle diameter [41], and struts near the nozzle diameter exhibit waviness [21]. For interfaces, the consistent qualitative findings are to prefer continuous (TPMS) or interlocked interfaces [79,94,96] and to avoid abrupt material transitions at strut nodes [6]. Quantitative interface-strength targets for graded lattices have not yet been established (Section 10).

**Table 5:** Design guidelines emerging from the reviewed studies.

Guideline	Refs.
Rigid–hyperelastic pairs give the largest energy-absorption gains, but the magnitude depends on configuration, baseline and loading rate	[6,16,36,47,49,50,51,93]
Weak immiscible interfaces, rather than the lattice, often limit strength and ductility, although interfaces stayed intact in several quasi-static compression tests	[6,11,49,52]; [23,35]
Density- or thickness-graded lattices collapse layer-wise; their energy gain over uniform lattices of equal mass depending on strain and is often small	[42,44,57,58,62,63,65,67,69]; [2]
Thickness grading outperforms size grading for specific energy absorption in strut lattices	[44,67]; [12]
TPMS and interpenetrating architectures delay interfacial debonding compared with strut nodes	[94,96]; [37]
Stiff materials suit stretching-dominated cells; hyperelastic materials suit bending-dominated cells	[46,85]
Depositing the higher-temperature material onto the lower-temperature one improves bonding	[35]; [20]
Loading transverse to the printing direction weakens lattices, so print orientation must be chosen with respect to the main load	[44,67,107]
Bending-dominated graded lattices are more strain-rate-sensitive	[44]
Minimum feature size is governed by nozzle diameter	[41]; [21]
A general optimal relative density or interface-strength target	Not established

Citations after a semicolon are supporting studies or reviews (Supplementary Table S1).

## 7. Applications of Multi-Material Functionally Graded Polymer Lattices

The mechanical performance of MM-FGLs, as reported in Section 6, was evaluated experimentally. In contrast, this chapter will discuss how that same functionality can be applied to four major areas of engineering and how we can determine whether the transition from specimen-level characterization to the functional performance level is supported by data or rests on assumptions.

To distinguish demonstrated from anticipated uses, each application below is classified as prototype-level (an MM-FDM or FDM graded-lattice prototype of the application has been built and tested), experimentally demonstrated at specimen level (relevant MM-FDM or FDM graded-lattice specimens have been tested, but no application prototype), or prospective (proposed on the basis of the trends in Section 6, or supported only by other processes). Table 6 summarizes this classification.

### 7.1. Energy-Absorbing and Impact-Mitigation Structures

Multi-material functionally graded lattices (MM-FGLs) are most prominent today for energy absorption and crashworthiness. The purpose of creating such structures is to absorb as much energy as possible while minimizing the maximum force exerted upon other structures. Most traditional uniform lattices experience catastrophic failure at some point during a crash event. Also, most lattices experience extremely high stress at the start of a crash, which can damage the protected payload. Using multi-material lattices that grade from a stiff thermoplastic (such as PLA or ABS) to a highly elastic polymer (such as TPU) allows designers to incorporate programmable collapse sequences [6,16,46].

Research has shown that using bio-inspired designs modeled after bone, specifically a dense cortex and a porous trabecular core, is very effective for absorbing energy. Specifically, research has indicated that designing a structure with both a stiff cortex and a soft interior creates a confinement effect. The stiff phase inhibits initial buckling, while the soft phase absorbs energy through viscous flow, resulting in a 2–3-fold higher SEA than would be obtained with a single phase [6,16]. For example, vertically graded lattices that contain both stiff and soft elastomers enable a tailored response to impact. At lower impact energy levels, the soft phase reduces shock loading, enhancing user comfort, and at higher impact energy levels, the stiff phase is engaged, preventing bottoming out [46].

Multi-material gradients also offer advantages over single-material solutions under repeated impacts. When subjected to multiple hits, lattices containing elastomer phases demonstrate greater recoverability and resilience than their rigid-phase counterparts due to plastic buckling or fracture [2,16,105]. Such recoverability would be relevant to multi-impact applications such as reusable helmet liners or bumper energy absorbers, which must regain their dimensions and energy-absorbing capacity after each impact. No helmet or bumper prototype based on MM-FDM graded lattices was identified in the reviewed studies, and the supporting evidence remains at the specimen level [16,44,105]; graded lattice-reinforced tubes produced by laser powder-bed fusion, an adjacent process, address the same requirement [109]. Note that the recoverability exhibited by 4D-printed SMP/hyperelastic systems [105], which relies on thermal activation, cannot be directly compared to that exhibited by TPU-based systems exhibiting passive elastic recoverability. That is because SMP/hyperelastic systems target recovery of functional performance over multiple cycles, whereas TPU-based systems target single-event damping, and their SEA values cannot be equated. A comprehensive survey conducted by Saber et al. [108] documented geometric design enhancements developed using AM for crash boxes that utilize gradient lattice infill patterns to meet automotive energy absorption standards while reducing mass compared to solid-walled counterparts.

### 7.2. Lightweight Structural Components

The need to reduce mass while preserving structural stiffness and strength motivates the use of FGL cores in sandwich panels and tubular structures, with aerospace and automotive components frequently cited as target applications. The evidence reviewed here is at the coupon and sub-component levels; no flight- or vehicle-qualified MM-FDM-graded component was identified. FGL cores made utilizing multi-material FDM to enable spatially tailored stiffnesses. High-modulus materials are placed only in locations along the load path, thus maximizing the strength-to-weight ratio [3,106].

Advances in radial functional grading have been found particularly useful for cylindrical shapes. Tubes having thin walls filled with radially graded multi-material lattices transitioning from soft centers to stiffer exterior layers demonstrate substantially enhanced crashworthiness and flexural stiffness as compared to uniformly constructed counterparts [3,61,106]. In an adjacent laser powder-bed fusion study, Wang et al. [109] showed that thin-walled tubes filled with double-gradient lattices that undergo dynamic impact exhibit superior crashworthiness metrics compared to single-gradient cores. The presence of a gradient alleviates the rapid stiffness discontinuity between the tube wall and the core, which delays local buckling and increases global energy dissipation. Graded sandwich cores have been produced by FDM through graded wall thickness [3,106], and speed-modulated printing grades core density without changing the toolpath [19]; the dynamic crushing of sandwich structures with different cell cores under impact has been characterized experimentally and numerically [118].

There exist, however, trade-offs in structural applications. While multi-material lattices can optimize certain properties, the addition of multi-material interfaces can create weaknesses if there is poor thermodynamic compatibility between the various materials. Research has shown that although functionally graded cores provide improved bending stiffness and delayed delamination, ultimately, the ability of the structure to bear loads is generally dependent upon the shear strength of the interface between the two incompatible polymers [44,61]. Therefore, to successfully implement structural elements composed of functionally graded cores, rigorous optimization of printing parameters must be performed to ensure that bond strengths at the interfaces between dissimilar polymers are sufficient to meet the lattice’s performance demands.

### 7.3. Vibration Damping and Stiffness Tailoring

Multi-material lattices are used increasingly as vibration dampeners and dynamically responsive structures. Because they are made from combinations of rigid and viscoelastic polymers, these lattices allow the separation of stiffness and damping characteristics that cannot be achieved simultaneously in one-piece monolithic parts. Multi-material designs involve a stiff matrix that contains areas of a softer phase. Engineers can vary the distribution of the softer phase to alter the structure’s frequency responses and loss factors [14,89,119].

Studies on experimental tests involving multi-material and foam-filled lattices show that when the weight percentage of a soft phase is increased significantly beyond what would be expected in a monolithic structure, the specific damping capacity (SDC) of those test specimens increases dramatically over that found in either an empty or an equal-weight single-material lattice [2,14,110]. The internal friction and interfacial slipping during cyclic loading promote energy dissipation through the interaction between the stiff skeleton and the soft filler [110]. The architecture of the lattice itself plays a crucial role; plate-type geometries enable a complete decoupling of the acoustic and mechanical properties of a material, allowing for the development of sound-absorbing metamaterials that are structurally rigid [60]. Potential uses include automotive interior panels and mounts for precision equipment, where lightweight structures both absorb vibration and reduce noise, although these have not yet been demonstrated with MM-FDM graded lattices.

### 7.4. Soft–Rigid Integrated Systems

The ability to create monolithic parts using multi-material FDM that contain multiple hard and soft regions has opened up new opportunities for creating classes of integrated devices for soft robotics, prosthetics, and wearable technology, where the structure interacts with the human body or environment via a functional gradation [9,73]. Polymer–ceramic graded scaffolds illustrate the same principle outside thermoplastic MM-FDM [120].

For medical applications, graded lattices provide a continuum of modulus values across interfaces, replacing abrupt changes from rigid to compliant states, which reduce the interfacial stresses present at sharp interfaces and protect adjacent tissues. Biomedical applications include patient-specific prosthetic liner designs based on varying ratios of ABS to TPU to replicate localized pressure distributions [31,73]; optimization frameworks, including topology optimization, FEA, and DoE, develop targeted stiffness in below-knee socket designs [111] and graded porosity insole designs that are optimized to achieve target deformation values corresponding to different stages of gait loading [41]. Lattice structures containing liquids further expand this concept: upon application of external force, the liquid contained within the lattice stiffens the soft skeletal structure; thereby replicating some aspects of intervertebral disc biomechanics [27].

Soft robotic applications utilize lattice skeletons with embedded inextensible elements to restrict radial growth, called “balloon effect,” while maintaining longitudinal stretching capabilities [9,115]; the example in [115] was produced by material jetting, an adjacent process cited for comparison. The same type of geometric organization enables 4D-printed actuators that consist of SMC layers paired with HEP layers to produce reversible, temperature-activated shape changing and load-bearing reconfigurations [105]. Single-material studies provide supporting evidence for the underlying shape-memory behavior: porous PLA–TPU blend structures [112], printed PVC [113], and re-entrant SMTPU strips whose recovery ratio can be varied as a function of temperature during extension [114]. Two adjacent studies are noted for comparison only: hierarchically structured scaffolds fabricated by electrospinning/extrusion [116] and hybrid bioprinting of tissue constructs with patterned biochemical signals [117].

## 8. Modeling, Simulation, and Design Tools

Functionally graded polymer lattice structures require computational models that integrate multiple scales to bridge the microstructure level detail with the macroscopic performance prediction. Due to process-induced anisotropy, non-uniformity of interfacial bonding strength, and discrete transition in material property, MM-FDM represents a challenge for classical solid mechanics modeling. Therefore, recent research activities focus on establishing robust homogenization methods, high-fidelity finite element models, and data-driven optimization tools for predicting and tailoring the behavior of those complex heterogeneous metamaterials.

### 8.1. Analytical and Homogenization Approaches

Homogenization approaches using representative volume elements (RVEs) offer an essential way to overcome the high computation cost when performing full-scale lattice structure simulations. The classical analytical scaling laws of Gibson & Ashby and those from other researchers remain important references for estimating the effective elastic moduli and yield strengths of lattice structures as functions of relative density [94,121]. However, analytical models are often limited to isotropic conditions and perfect geometry reproduction. Thus, they may fail to adequately predict the directionally dependent stiffnesses found in FDM-fabricated lattices and also the complex stress states encountered in graded topological structures [15,76].

As a result, many researchers are employing numerical homogenization under periodic boundary conditions (PBCs) to calculate the effective elastic tensors that account for the layer-wise orthogonal nature of FDM deposition [76]. One of the most significant developments in this field has been the incorporation of manufacturing factors within the homogenization approach. Many researchers have demonstrated that incorporating FDM fabrication parameters, such as layer thickness, raster angle, and air gap spacing, into the RVE model improves the accuracy of predicted effective structural properties compared with those calculated using isotropically assumed simple models [76,84]. For example, Nguyen et al. [107] provided a digital fabrication AM method that directly embedded anisotropy induced by FDM processing into a density variable, effectively validating the methodology through three-point bending tests. Physics-augmented neural networks, i.e., neural-network surrogates constrained to satisfy thermodynamic consistency, were introduced to provide a scalable method for continuous grading of structures without requiring recomputation of RVEs at each density iteration. Shojaee et al. [15], working with stereolithography-printed shell lattices (an adjacent process), showed how these networks could be applied to map geometric parameters to homogenized elasticity tensors while maintaining thermodynamic consistency and greatly improving calculation speed by orders of magnitude compared to direct finite element analysis. Although great strides have been made in this area, challenges remain regarding scalability. Specifically, homogenizing multi-material interfaces where discrete property changes occur can require complex asymptotic expansions that typically demand extensive computational resources [15].

### 8.2. Finite Element Modeling of Multi-Material Lattices

FEA is used primarily to verify the effectiveness of multi-material lattice structures. However, FEA comes with a tradeoff between computational efficiency and geometric fidelity. Beam-based finite element models are economically viable for large-scale truss-based lattice structures and are commonly used [60,83]. However, because beam-based models do not accurately represent stress concentrations at strut intersections and node overlap points, they tend to overestimate compliance and delay failure due to densification [90,122]. Therefore, second-order tetrahedral solid elements are used to simulate multi-material lattice structures with complex deformation mechanisms and contact interactions, but they incur additional computational expense [60].

Accurate representations of interfaces between different materials in multi-material FDM structures are critical. Perfect-bond assumptions in FEA tend to overestimate stiffness, peak load and energy absorption, because they neglect the weak diffusion bonding between thermodynamically incompatible polymers [123]. Recently advanced modeling methodologies have incorporated cohesive zone models (CZMs), linear elastic fracture mechanics (LEFMs), or maximum tangential stress (MTS) criteria to simulate interface debonding and mixed-mode fracture between dissimilar polymers [123,124]. Angle-dependent constitutive models have been developed to represent the layer-wise anisotropy of FDM, assigning material properties to struts based on the printing orientation relative to the build platform [89,107,125]; the same approach has been applied in an adjacent vat-photopolymerization study [80]. In addition, validation practices in the literature increasingly advocate representing manufacturing defects such as strut waviness, surface roughness, and variable cross sections in FE models, for example via Monte Carlo simulation, so that simulated results correspond to measured quasi-static and dynamic compression data [21,89,107].

### 8.3. CAD, Voxel-Based, and Gradient-Mapping Workflows

Traditional B-Rep CAD methods for FGLs have failed to represent their geometric complexities efficiently. The file sizes produced by B-Reps and Boolean operations limit the effectiveness of the B-Rep method [14,84]. Therefore, implicit modeling is an efficient method for developing spatially varying architectures. Specifically, it was demonstrated that level-set methods and TPMS are suitable alternatives to B-Reps for developing FGLs [76,97]. As an additional benefit, the implicit modeling method provides a means to achieve functional grading by modulating either the isovalue or the thickness parameter of the implicit function using mathematical fields. This modulation method provides smooth transitions between different densities or cell configurations that would otherwise cause mesh errors in STL files [60,76].

An important development in voxel-based and direct G-code generation workflows was enabled by the work of Grigolato et al. and Gleadall [81,84], who proposed a method to bypass the STL file format entirely and use volumetric density maps to modify G-code instructions directly. Since the volumetric density map method enables the variation of infill density and shell thickness at the machine level, it eliminates the “sliced-CAD” limitations that cause gradient information to be lost during fabrication [1,19]. Voxel-based representation also enables the discrete assignment of different materials to specific spatial coordinates in multi-material settings. Such voxel-based representations enable the design of complex IPCCs and bio-inspired gradients that are not achievable with standard surface meshes [15,60,93]. Finally, Full Control G-Code Designer and other open-source tools have provided “process-driven” grading techniques. Process-driven grading involves dynamically modulating extrusion width and speed along the tool path to produce functional gradients that do not exist in the CAD geometry [126].

### 8.4. Optimal Design Methods for FGL Materials

Optimization frameworks for multi-material lattices have progressed significantly from basic parameter sweeps to complex TO and machine learning (ML)-based designs [73,127]. Density-based TO methods, such as solid isotropic material with penalization (SIMP), are commonly used to optimize material distribution to maximize stiffness or energy absorption [84]. It was shown by Hassani et al. [128] that explicit multi-material composition optimization results in better structural performance than single-material SIMP under flexural loading conditions on FDM specimens when the ratio of modulus between the phases is high. Concurrent optimization methods have been developed to simultaneously optimize both the macro-scale material distribution and the micro-scale lattice configuration to improve overall structural performance, as demonstrated in an adjacent stereolithography study [127].

Recent developments in data-driven optimization address the multi-objective nature of lattice design, i.e., conflicting objectives such as stiffness, weight, and energy absorption [129]. A combination of genetic algorithms (e.g., NSGA-II) and neural networks as surrogate models has proven effective for finding optimal solutions in the vast design space of truss and TPMS lattices while greatly reducing the number of required FEA evaluations [129]. Generative deep-learning models have also been applied to lattice design. Graph neural networks have been used for the numerical multi-objective design of multi-material truss lattices [122], and a latent diffusion model has been used to generate new single-material cellular structures [121]. Neither has been demonstrated experimentally for MM-FDM graded lattices yet, so their use here remains prospective. The data-driven approaches with experimental MM-FDM evidence are narrower: neural-network surrogates trained on printed PLA/TPU TPMS specimens [16], inverse design of multi-material gyroids [93], and deep transfer learning for the properties of dual-material FFF composites [130]. Few current optimization frameworks include MM-FDM manufacturing constraints such as nozzle-switching overhead, purge waste or interface strength [20,28,125].

Table 7 compares these approaches on a common basis. The validation column refers only to experimental validation reported for FDM or MM-FDM lattices in the reviewed studies; many of the methods are, in general, more mature in lattice mechanics than their MM-FDM record shows. Cost ratings are qualitative and relative.

## 9. Manufacturing Constraints and Current Challenges

Although MM-FDM has tremendous potential to enable designers to fabricate functionally graded polymer lattice structures that exhibit unique combinations of mechanical properties across multiple length scales, the transition from prototype demonstrations in research laboratories to scalable industrial production is currently limited by numerous physicochemical and technological constraints. Although the ability to control material properties along three dimensions offers designers unprecedented flexibility in creating structures with spatially varying properties, limitations remain in terms of how effectively the physical process can replicate these variations. This section presents a review of major obstacles facing those seeking to scale up multi-material FDM-based fabrication processes.

### 9.1. Material Compatibility and Interfacial Reliability

As established in Section 6.4, multi-material FDM lattices combining thermodynamically incompatible polymers suffer from weak diffusion bonding governed by wetting behavior and solubility parameter mismatch, producing interfacial stress concentrations that cause delamination prior to the onset of the designed collapse mechanism. This failure mode is especially pronounced in hard–soft combinations; full mechanistic characterization is provided in Section 6.4 [17]. Since the bond strength at the interface is determined primarily by the wetting behavior and solubility parameters of the polymers involved, large discrepancies in these parameters result in weak adhesive contacts and subsequent delamination of the layers under loading [20,29]. This is especially important for lattice designs using “hard–soft” combinations, since the interfaces represent a sudden drop in stiffness, and therefore act as stress concentration points when subjected to mechanical loads. Prior to the onset of progressive collapse of the lattice struts under the applied mechanical load, failure will occur at the interface [6,11,20,29].

In addition, there are problems associated with thermal mismatches. Because the sequential deposition of materials with different coefficients of thermal expansion (CTEs) causes residual stresses upon cooling, this leads to warpage and interfacial shear fractures [119]. Although certain research indicates that mechanical interlocking through complex path programming can mitigate these issues, there remains a lack of chemical affinity among common engineering thermoplastic materials, preventing the attainment of bulk-equivalent strengths [79,110]. The long-term stability of these interfaces under fatigue or environmental loading conditions is poorly understood, raising concerns about the durability of graded lattice structures in operational environments [4,5,20,28].

### 9.2. Process-Induced Defects and Print Reliability

The reliability of multi-material FDM is often compromised by the hardware limitations of extrusion systems. The most prominent cause of defects is material cross-contamination. For example, in a single nozzle mixing system or during the transition from one nozzle to another in multi-extruder systems [5,20,28,35], inactive nozzles tend to ooze, causing unwanted residue on lattice struts, affecting local mechanical properties, and increasing surface roughness [5,18,21]. Contaminants from cross-contamination can plug pores or introduce unintended stress raisers, decreasing fatigue life in porous lattice architectures with fine feature sizes [89].

Because of the inherent “start–stop” action in depositing non-continuous struts in lattice structures, additional voids may form. Retraction and re-prime actions of nozzles typically result in incomplete extrusions or air entrapped at nodes in the lattice, which are key locations for load transfer [20,21,28]. Voids generated during processing differ from intentionally created porosity and significantly decrease both the modulus and strength of the structure relative to theoretical values [20,21]. Poor coordination between material flow rates and printer head movements during transitions between materials can also generate cold joint weaknesses or separate seams that serve as crack-initiating sites. The random occurrence of these defects makes predicting reliable mechanical responses of multi-material lattices extremely challenging and consequently requires high safety margins that diminish the weight-reduction capabilities of lattice designs [4,20,119].

### 9.3. Geometric Accuracy and Resolution Limitations

Dimensional fidelity of FDM is fundamentally limited by the nozzle diameter and the rheological characteristics of the molten polymer, resulting in a trade-off between grade resolution and printability [131]. Struts formed in lattices with dimensions close to nozzle diameters exhibit noticeable geometric errors, including wave patterns along the length of the struts and variability in cross-sectional areas [21]. Recent experimental studies have shown that low-relative-density lattices are particularly sensitive to failure during processing; thin, fragile struts do not possess enough structural integrity to resist forces exerted by the moving nozzle during the deposition process, causing cracking or severe deformation [21].

The limitation described above is particularly relevant to functionally graded structures in which the struts vary continuously in thickness or unit cell size. Current slicing software generates “stepwise” gradations instead of the desired continuous gradations, resulting in a “stair-step” deviation from the designed smooth curvatures, particularly for surface-based TPMS architecture [14]. In addition, controlling flow rates to produce gradient densities (process-induced grading) is further complicated by the viscoelastic time delay of the polymer melt, resulting in a discrepancy between the commanded and the actual deposited material [14]. Deviations from the intended geometry create discrepancies between the as-designed CAD model and the as-fabricated part, complicating the verification of finite element models and the certification of parts for aerospace and biomedical applications [4,21].

### 9.4. Scalability and Industrial Adoption Challenges

Additive manufacturing is less expensive than other technologies for making prototypes. However, many technical challenges must be overcome to produce large numbers of multi-material functional parts economically. For example, it is computationally intensive to generate the slices necessary to fabricate complex FGLs because the algorithm must produce numerous tool paths and perform numerous material changes per million unit cells [19,21]. In addition, physically, there is a limit to how fast you can print due to both the heat generated by the polymer(s) being deposited and the mechanical inertia of the head of the printer, especially when dealing with multiple nozzle systems, because changing tools will add significant time to the total build time [28,131].

In terms of quality control, non-destructively inspecting internal defects within multi-material latticed parts is very difficult. Techniques such as X-ray microcomputed tomography (μ-CT), while effective for determining whether there are internal defects, take too long and cost too much to be practical for in-line quality control in a manufacturing facility [4,20]. The absence of robust in situ monitoring systems that can identify and correct inter-material defects in real time severely limits the ability to achieve high levels of process reliability, critical for high-value applications [4,28]. Finally, the energy consumption created by heated chambers and multiple extruders creates a sustainability issue that must be solved prior to achieving widespread industrial acceptance [20,35].

## 10. Research Gaps and Future Directions

It will be challenging to transfer multi-material FGLs from research laboratory demonstrations to commercial production due to interconnected technological barriers, including limitations in the functional grading strategies employed during the additive fabrication process; limitations in the capabilities of the existing hardware used for the additive fabrication processes; predictive modeling deficiencies; and a lack of end-of-life management technologies. The next section describes the unresolved technical challenges associated with transitioning MM-FGLs from laboratory demonstrations to commercial applications and presents strategic areas for future research to address them.

### 10.1. Technical Challenges Associated with Current Functional Grading Techniques

Currently, functional grading in polymer Fused Deposition Modeling has been achieved primarily through discrete and incremental techniques. These discrete and incremental techniques have limited similarity to the continuous graded properties found in nature. The majority of multi-material lattices currently being developed utilize zone-based approaches, in which individual zones are created from a particular material with a defined property, and multiple zones are then combined. Zones represent significant discontinuities in both the material properties and the lattice structure, thereby acting as sites of localized stress concentrations. Often, the introduction of a zone leads to rapid lattice degradation due to the initiation of failure at the boundary between two distinct materials. Although it is theoretically possible to achieve continuous functional grading using active mixing nozzles, there are significant practical difficulties in producing a continuous transition between two commonly used thermoplastics. For example, developing a seamless transition between semi-crystalline and amorphous-phase thermoplastics such as PLA and TPU, respectively, represents a significant challenge due to their differing melting temperatures and viscosities.

Therefore, future development efforts related to MM-FGLs should focus on developing process-driven grading techniques that enable modulation of composition at the voxel level, using active mixing extruders to enable in situ blending of thermoplastics with significantly disparate rheologies. Additionally, the study of functional grading techniques focused solely on stiffness grading may need to expand to include multifunctional gradient techniques that create simultaneous conductivity, electromagnetic shielding, or thermal control functions within the same part.

### 10.2. Multi-Material FDM Hardware and Process Control

Presently, existing hardware is considered the primary barrier to the manufacturing of scalable MM-FGLs. Specifically, dual nozzle and tool changing systems experience problems with oozing, contamination of each other’s output streams, synchronization errors resulting in stringing or cold welds in thin struts, longer processing times due to the start/stop nature of material changes and thermal cycling that reduces layer-to-layer adhesion. Many combinations of thermodynamic incompatibilities between polymers limit the number of materials that can be utilized in combination. Nozzle, bed and chamber temperatures, switching time and purge volume are seldom varied or reported as controlled factors in the reviewed lattice studies (Table 2); reporting them routinely would make interfacial performance comparable across studies.

The next generation of multi-material platforms would benefit from convergence toward multi-material single-nozzle (MMSN) and hybrid configurations, as well as liquid-entrained extrusion (LEE) with active in-line mixing, enabling continuous material change without requiring tool changes. Also, the incorporation of in situ polymerization baths, as well as auxiliary energy sources (e.g., laser or microwave heating), could enable access to additional chemistries beyond thermoplastics, e.g., thermosets. Finally, ML- and vision-based closed-loop defect correction are required to achieve the geometric accuracy demanded by high-performance metamaterials.

### 10.3. Modeling, Simulation, and Design Gaps

There is still a significant gap between as-designed and as-manufactured performance. The standard FEA technique assumes idealized geometries and perfect bonding between components, systematically overestimating stiffness and energy absorption in systems where diffusion bonding is weak (see Section 6.4) and where strut waviness and build-direction anisotropy are present. Therefore, FEA tends to predict higher stiffness and impact resistance than those of actual manufactured parts. Slicers currently treat multi-material parts as collections of shells (discrete layers) rather than as continuous volumetric fields; therefore, they do not support volumetric AM design with true graded properties.

Physics-informed multiscale simulation tools will be needed. Asymptotic homogenization that explicitly accounts for interface conditions and manufacturing-induced defects provides a method for obtaining accurate macroscopic predictions. Digital twin models that simulate all aspects of temperature and deposition histories during manufacture can be used to inform optimized tool paths and reduce residual stresses.

### 10.4. Data-Driven and Optimization-Based Design

The design space for multi-material FGLs is sufficiently large that traditional TO methodologies fail to simultaneously optimize both material distribution and unit cell geometry under manufacturing constraints. Data-driven design methodology can potentially overcome this limitation.

Generative deep learning models such as graph neural networks (GNNs) and latent diffusion models (LDMs) have shown in numerical and single-material studies [121,122] that they can propose architectures beyond conventional lattice libraries. Extending them to MM-FDM graded lattices would require training data that capture interface strength and process-induced defects, and experimental validation on printed multi-material specimens, neither of which is yet available. To realize this potential requires creating standardized open datasets containing experimental and simulated mechanical testing data for lattice structures, so that validation studies can move away from ad hoc comparisons and towards physics-informed evaluations.

### 10.5. Sustainability, Recyclability, and Lifecycle

As MM-FDM technology adoption increases, it will become increasingly important to consider the sustainability implications of creating composite structures from chemically distinct polymers or embedded conductive filler particles that cannot be easily separated at end-of-life, thus violating linear lifecycle principles inherent to most sustainable product designs.

Two potential solutions to this issue include bio-based graded composites, such as PLA variants containing natural fiber or food waste reinforcement, and reversible bonding mechanisms/stimuli-responsive interfaces enabled by 4D printing, enabling the on-demand separation of materials at end-of-service life.

## 11. Conclusions

This review synthesized functionally graded polymer lattice structures produced using multi-material fused deposition modeling (MM-FDM) according to the four grading methods discussed above (material-based zoning, geometry-induced grading, combined material–geometry grading and process-induced grading) and assessed each of the grading methods according to a variety of topological geometries (strut-based, TPMS, and hybrid plate–TPMS). The study’s focus was limited to thermoplastic MM-FDM, a narrower scope than prior reviews, allowing closer attention to how FDM-specific processing limitations influence the implementation of the grading method. The conclusions below rest on the 65 core studies identified by a non-exhaustive search; supporting and adjacent studies were used only if labeled as such.

The major conclusion is that combinations of rigid thermoplastics (PLA, ABS, PETG) and highly elastic TPU, in either core–shell or interpenetrating phase configurations, are the most effective pairings documented so far, with reported increases in specific energy absorption of up to two- to threefold over single-phase counterparts, mainly through confinement and progressive collapse; the size of the gain depends on configuration, on the baseline chosen and on loading rate, and against the rigid constituent alone the quasi-static gains were small or negative in several studies. Geometry-induced grading provides a separate means of controlling the failure sequence, although its energy gain over uniform lattices of equal mass was strain-dependent and often modest; in the strut-based comparison reported, thickness grading outperformed size grading in energy absorption. A combination of material and geometry grading allows designers the greatest degree of freedom to optimize their design but requires sophisticated computational capabilities to address interactions between material interfaces and geometric gradients. Gradients achieved through process-induced methods include print speed modulation and the incorporation of thermally expandable microspheres, which allow grading without modifying the CAD geometry, at the cost of a less predictable response. These findings are collected as design guidelines in Table 5; because the underlying studies differ in material, topology and test conditions, the guidelines should be treated as trends until they are confirmed under common test protocols.

From a topological perspective, TPMS architectures have structural advantages due to continuous, self-supporting interfaces, which reduce stress concentrations at nodes associated with delamination of interfaces between different materials in strut designs and enhance in situ thermal bonding between dissimilar phases in interpenetrating configurations. Hybrid plate-topology periodic media lattices, the newest member in this family of architectures, have been reported to provide enhanced tensile-dominated stiffness and improved interface continuity compared with strut and surface topologies alone. From a computational standpoint, the field is moving from isotropic finite elements and B-rep CAD towards voxel-based implicit workflows, process-aware homogenization and data-driven surrogates. Of these, only process-aware homogenization and surrogate models have been validated to date against printed FDM or MM-FDM specimens.

Most of the evidence remains at the specimen level. Of the applications discussed in Section 7, only a multi-material prosthetic socket has reached prototype level, and uses in helmets, vehicles and aircraft remain prospective (Table 6). Three challenges hinder the transfer of these laboratory results to industrial applications. Weak diffusion bonding between thermodynamically incompatible polymer pairs often leads to failure by delamination before the planned collapse mechanisms are engaged. Defects introduced by processes (nozzle oozing, cold joint formation, etc.) result in a stochastic relationship between as-designed and as-fabricated performance that current predictive models are unable to capture since they assume perfect bond quality and idealized geometry. Finally, no standardized test protocols exist for comparing graded heterogeneous structures across studies or for validating the models developed to simulate them.

Addressing these challenges will require compatibilizers and active mixing extruders that can produce continuous, viscosity-matched gradients between incompatible polymers; multiscale models that carry fabrication-induced defect distributions into macroscale predictions; and agreed characterization standards that account for the anisotropy and gradient complexity of graded multi-material lattices.

## Figures and Tables

**Table 1:** Search strings used for literature retrieval.

ID	Search String
S1	(“functionally graded” OR “functional grading” OR “graded lattice”) AND (“lattice structure” OR “cellular structure”) AND (“multi-material” OR “dual material” OR “dual extrusion”) AND (“fused deposition modeling” OR “FDM” OR “material extrusion” OR “FFF”) AND (“polymer” OR “thermoplastic”)
S2	(“lattice structure” OR “TPMS lattice” OR “cellular lattice”) AND (“multi-material” OR “dual extrusion”) AND (“FDM” OR “fused deposition modeling”) AND (“polymer”)
S3	(“multi-material lattice”) AND (“energy absorption” OR “crashworthiness”) AND (“FDM”) AND (“polymer”)
S4	(“multi-material FDM”) AND (“interfacial bonding” OR “interface strength”) AND (“lattice”)
S5	(“multi-material” OR “multimaterial” OR “dual material” OR “dual extrusion”) AND (“lattice structure” OR “cellular structure”) AND (“fused deposition modeling” OR “FDM” OR “material extrusion” OR “FFF”) AND (“polymer” OR “thermoplastic”)

**Table 2:** Process parameters in MM-FDM of graded polymer lattices: mechanism and reported effect.

Parameter	Mechanism	Reported Effect	Refs.
Nozzle temperature	Melt viscosity; interdiffusion time	Varied in one lattice study, where it changed the compressive modulus; otherwise reported only as a fixed setting	[41]; [6,16,36]
Bed/chamber temperature	Cooling rate; residual stress	Bed temperature reported as a fixed setting; chamber temperature not reported	[6,16,36,42]
Print speed/extrusion rate	Deposited bead volume	Speed modulation produces density grading from an unmodified toolpath	[19]
Infill density	Local solid fraction	Stiffness gradation without changing the toolpath	[43]
Layer height	Interlayer contact; stair-stepping	Affects compressive modulus, print time and dimensional error	[41]
Nozzle diameter	Minimum strut size	Affects modulus and dimensional error; struts near the nozzle size show waviness	[41]; [21]
Raster/build orientation	Interlayer anisotropy	Lattice response depends on the loading direction relative to the print direction	[44]; [45]
Material switching	Pressure lag after a change	Under-extrusion or blobs at interfaces	[23]; [20]
Purge (flushing) volume	Clears the previous material	Controls PLA/PETG interface behavior (non-lattice)	[33]
Interfacial temperature history	Re-melting at the dissimilar interface	Deposition order affects bonding; cold bonds when reversed	[35]; [20]

Citations after a semicolon are supporting studies or reviews (Supplementary Table S1). Ref. [33] is not part of the screened set.

**Table 3:** Comparison of functional grading strategies for multi-material FDM polymer lattice structures.

Strategy	Grading Mechanism	Typical Implementation in MM-FDM	Reported Performance Benefit	Key Limitation	Refs.
Material-based (discrete zoning)	Spatial variation in polymer composition between distinct lattice regions; typically pairs a stiff thermoplastic (PLA, ABS) with a hyperelastic elastomer (TPU).	Discrete tessellation via dual-nozzle or tool-changing FDM; rigid-shell/soft-core struts; bio-inspired cortical–trabecular analogs.	SEA is 2–3 times higher than that of single-phase counterparts via the confinement effect; it deflects stress paths, converting catastrophic shear into progressive, layer-by-layer collapse.	Sharp interfaces concentrate stress; thermodynamic incompatibility between dissimilar polymers yields weak diffusion bonding and premature delamination.	[6,9,11,15,21,36,37]
Geometry-induced (thickness/size grading)	Spatial variation in relative density via strut thickness or unit cell size at constant composition; may include topology transitions (e.g., stretching- to bending-dominated).	Thickness-graded or size-graded strut and TPMS lattices; gradient profiles aligned with the loading direction; smooth stiffness transitions across layers.	Decouples local stiffness from mass; enables layer-wise progressive collapse and elongated plateau stress; thickness grading typically outperforms size grading for SEA in strut lattices.	Density reductions inherently lower strength; coupled stiffness–strength trade-off cannot be resolved by geometry alone; stepped transitions introduce staircase artifacts.	[2,11,42,44,45,46,58,59,61,82]
Combined material–geometry grading	Concurrent modulation of material composition and geometric density, allowing local stiffness and topology to be tuned independently.	Vertically stacked cells with varying plate thickness and alternating reinforced/neat polymer layers; radially graded BCC/FCC/Octet hybrids; interpenetrating-phase TPMS composites.	Surpasses single-parameter grading; programs the layer-collapse sequence; stiff outer layers confine softer cores, suppressing global buckling and enforcing progressive plastic deformation.	Requires advanced multi-scale modeling to capture interface effects, residual stresses, and Poisson-ratio mismatch; manufacturing complexity rises with each added gradient axis.	[14,35,42,45,57,60,61,75,82]
Process-induced (parameter-driven)	Modulation of FDM process parameters: extrusion speed, flow rate, infill density, raster angle to vary local density without altering the CAD geometry.	Speed-modulated printing of graded TPMS from uniform STL; gradient infill percentage; interlaced or topologically interlocked dual-material toolpaths; thermally expandable microspheres in TPU.	Streamlines workflow (no CAD/STL modification); enables continuous multi-level microcellular gradients; allows filament alignment with principal stress directions; mechanical interlocks improve delamination resistance.	Viscoelastic lag causes a mismatch between commanded and actual deposition; the nozzle diameter bounds resolution; mechanical response is harder to predict and certify.	[17,23,28,32,43,78,79,80,81,83]

**Table 4:** Test conditions and mechanical outcomes of selected core studies with experimental data: (a) multi-material FDM lattices; (b) single-material, geometry-graded FDM lattices.

Ref.	Materials (Ratio)	Topology; Grading	Relative Density/Infill	Specimen	Loading (Rate; Direction)	Print Orientation; Nozzle	Exp./Num.	Key Finding
**(a) Multi-material FDM lattices**								
[6]	PLA shell/TPU 95A core; core-to-strut thickness ratio c/t 0–1	Planar hexagonal honeycomb; soft-core/hard-shell struts	≈32%	10 × 44 mm footprint, 46 mm high	In-plane quasi-static compression, 2 mm/min (≈7 × 10^−4^ s^−1^) *	XYZ orientation, rasters along struts; NR	Exp. + Num. (FE, cohesive zone)	Energy absorption ≈2–3 times that of the single-material constituents; all lattices failed at the PLA–TPU interface
[16]	PLA/TPU; 0, 25, 50, 75, 100% PLA	Diamond TPMS (15 mm cells); material distribution	40, 50, 60%	30 × 30 × 30 mm	Quasi-static compression with unloading; rate NR	NR; NR	Exp. + ML (ANN)	Modulus, energy absorption and peak load rise, and energy loss fall, with relative density and PLA fraction; ANN-designed lattices met targets in tests
[36]	ABS/TPU 95A; central TPU band 0–12 mm high	Square and hexagonal honeycombs; material band	NR (designs within 5% mass)	ASTM D1621 sizing (source Table 1)	Compression, 100 mm/min (0.13 s^−1^); out-of-plane and in-plane	NR; 0.4 mm	Exp.	Out-of-plane EA 2.2–11.5 kN·mm (square) and 2.9–15.1 kN·mm (hexagonal) from TPU to ABS; in-plane 0.1–2.6 and 0.6–2.0 kN·mm
[77]	Porous TPU/plain TPU/CF-TPU; up to three zones	Honeycomb (5 mm cells, 1.2 mm walls); out-of-plane material gradient	NR	70.69 × 51.96 × 25 mm	Out-of-plane compression, 0.025 s^−1^	Gradient along build direction: 0.4 mm	Exp. + Num. (FE)	CF-TPU absorbed most energy above ≈4.5 MPa and porous TPU below ≈1 MPa; graded honeycombs balanced absorption over a wider stress range
[93]	PLA/TPU; TPU:PLA 3:1, 1:1, 1:3	Gyroid (sheet TPMS); material ratio and relative density	40, 50, 60%	30 × 30 × 30 mm	Uniaxial compression to 50%, 0.025 mm/s (≈8 × 10^−4^ s^−1^) *; along z	Loaded along z; NR	Exp. + ML (NN)	Interpolation captured density–ratio–property relations; NN inverse design predicted target modulus, EA and peak stress, confirmed by tests
[42]	Tough Nylon/Onyx (short-CF nylon), layer-wise	Octet plate-lattice; plate-thickness grading and material layering	NR (plates 1.0–2.0 mm)	NR	Quasi-static compression, 10 mm/min; along grading direction	Grading along the build/load axis: 0.4 mm	Exp. + Num. (FE)	Thickness-graded designs ≈10% higher SEA at large strain; material arrangement shifted the onset of collapse
[105]	FlexPro elastomer/polyurethane-based SMP	Re-entrant auxetic meta-sandwich cores; soft/hard combinations	NR	NR	Indentation compression with unloading; rate NR	Raster 0°; NR	Exp. + Num. (FE)	Tunable nonlinear stiffness and hysteresis; plastic deformation fully recovered by heating
[47]	PLA matrix/TPU in stress-concentration rows; 6.7–29.4 vol% TPU	Schwarzite-inspired porous structures; radial pore-size gradient and targeted TPU placement	NR	NR	Quasi-static compression, 1 mm/min	NR; NR	Exp. + Num. (FE, MD)	SEA up to 2.4 times that of the PLA-only and 4.9 times that of the TPU-only structure; one soft layer was optimal
[48]	PLA matrix/TPU in stress-concentration regions; 12, 24, 36% TPU	Zeolite-inspired (ATN, BEC) porous structures; stress-targeted TPU placement	NR	NR	Compression, 2 mm/min	NR; NR	Exp. + Num. (FE, MD)	Specific resilience up to 1.8 (ATN) and 3.2 (BEC) times that of PLA-only; 36% TPU performed worse than 24%
[49]	PLA/TPU; inner or outer sheet layer; TPU network 6:10–9:10	Primitive TPMS sheet (12.5 mm cells); material zoning through the wall thickness	27.5% (single); 55.4% (dual); 32.9–39.2% (network)	4 × 4 × 4 cells; bending beams 200 × 25 × 12.5 mm	Compression, 0.75 mm/min (10^−3^ s^−1^); three-point bending, 0.5 mm/min	NR; 0.4 mm	Exp. + Num. (FE)	SEA 12.4–13.4 J/g vs. 8.7 (PLA) and 2.9 J/g (TPU), at twice the PLA relative density; TPU-network design 19.2 J/g; interfaces intact
[50]	PLA/TPU assigned by strut family	Sea-sponge-inspired 2D lattice; material assignment by strut family	41%	60 × 60 × 20 mm	Quasi-static compression, in-plane and out-of-plane; rate NR	NR; 0.2 mm	Exp. + Num. (FE), DIC	Dual-material SEA 0.66–0.80 times that of PLA-only and 2.5–2.7 times that of TPU-only; steadier plateau and higher efficiency; no in-plane debonding
[51]	PETG/TPU; TPU outer honeycomb, PETG tube bundles	Biomimetic straight-wall and corrugated honeycombs; material zoning	≈0.20	48.5 mm diameter, 50 mm high	Axial quasi-static compression, 5 mm/min; impact, 14.9–26.6 m/s	NR; NR	Exp.	Quasi-static: PETG-only SEA 2.1–3.2 times the dual-material value; impact: dual-material SEA 35–66% higher, with layer-wise collapse
[52]	PLA+/PETG; 50/50 halves or PLA–PETG–PLA layers	Gyroid, diamond and primitive sheet TPMS; material layering along the build axis	0.3	30 × 30 × 30 mm (3 × 3 × 3 cells)	Quasi-static compression, 20 mm/min; along the build direction	Load along build direction; NR	Exp. + Num. (FE)	Single-material diamond PLA absorbed the most energy (≈128 J); no interfacial failure was observed
[55]	PLA/wood-filled PLA	Gyroid (PLA) and Schwarz-P (wood–PLA) regions; material and cell-type grading	NR	25 mm cube	Quasi-static compression; rate NR	NR; NR	Exp.	Hybrid SEA 1.1 and 1.8 times that of the uniform PLA gyroid and wood–PLA Schwarz-P lattices; strength 10.33 MPa
**(b) Single-material, geometry-graded FDM lattices**								
[97]	PLA	Gyroid (10 mm cells); wall thickness graded, 1–5 mm series	NR	50 × 50 × 50 mm	Quasi-static compression; rate NR	Vertical; NR	Exp.	Initial peak stress rose from 6.8 MPa (1 mm) to 25.3 MPa (5 mm series)
[61]	PLA	Radially hybridized beam (BCC, FCC, octet) and TPMS (diamond, gyroid, split-P) lattices; radial grading	Per design (source Table 1)	Cylinder, 54 mm high, three concentric zones	Quasi-static compression, 2 mm/min	NR; 0.4 mm	Exp. + Num.	Surface-based graded lattices gave the largest gains: modulus +71%, strength +36%, SEA +19%
[44]	Nylon or short-CF nylon (Onyx), one material per specimen	Surface- and strut-based lattices; density and cell-size grading	Mean 0.5	20 mm cube (4 × 4 × 4 cells)	Drop-weight impact, 15–90 J (22–79 s^−1^); parallel and transverse to print direction	Both directions tested; NR	Exp.	Graded lattices absorbed ≥15.2% more energy than ungraded; fiber reinforcement added up to 37.5%
[57]	Short-CF nylon (Onyx)	BCC and Schwarz-P; linear density grading (0.8–0.2, 0.7–0.3, 0.6–0.4)	Mean 0.5	30 × 30 × 30 mm (6 × 6 × 6 cells)	Quasi-static compression, 0.5 mm/min (≈3 × 10^−4^ s^−1^) *	Build direction along grading; NR	Exp. (DIC)	Grading changed energy absorption and deformation differently for BCC and Schwarz-P lattices of equal mass
[67]	Short-CF nylon (Onyx)	BCC, Schwarz-P and gyroid; density grading and cell-size grading (companion study to [57])	Mean 0.5	30 mm cube (6 × 6 × 6 cells)	Quasi-static compression; parallel and transverse to build	Both directions; ≈0.36 mm	Exp.	Graded Schwarz-P outperformed ungraded only beyond ≈46–51% strain; graded BCC was worse when loaded parallel; cell-size grading had no effect
[62]	TPU	Hexagonal honeycomb; 2-, 3- and 5-stage wall-thickness grading (1–5 mm)	Equal volume to uniform 3 mm walls	7 cells, 18 mm high	Out-of-plane compression, 5 mm/min	Along build direction: 0.4 mm	Exp.	Graded designs absorbed more energy than the equal-volume uniform design only at strains above 0.5
[63]	PLA	Hexagonal and triangular honeycombs; layer-wise wall thickness 0.6–1.0 mm	Equal mass (44 g)	≈47 × 50 × 50 mm, five layers	In-plane compression, 5 mm/min	NR; 0.4 mm	Exp. + Num. (FE)	SEA 2.5–21.2% higher than the equal-mass uniform honeycomb
[65]	PLA	Auxetic double-arrowhead lattice (5 × 5 cells); unit-cell angle grading	NR	20 mm deep, 28–57 mm high	In-plane compression, 1.5 mm/min	Rasters along load: 0.4 mm	Exp. + Num. (FE)	Best graded design (FE) SEA +173% vs. the uniform 10° design and +1.4% vs. the best uniform design
[64]	PLA, glass- or carbon-fiber PLA (one per specimen)	Rangoli-inspired cellular structure; cell densification at the faces or the center	0.65 (reported)	50 × 50 × 30 mm	Flatwise compression (ASTM C365), 2 mm/min	NR; 0.4 mm	Exp.	Carbon-fiber PLA: SEA 2.95 (center-densified) and 2.20 kJ/kg (face-densified) vs. 2.81 kJ/kg (base)
[68]	PLA	Bone-inspired 2D cellular structure; layers of compliant and stiff cell types	NR	20 mm cells, 20 mm deep	In-plane compression, 5 mm/min	Along struts; NR	Exp. + Num. (FE)	Hybrid lattice: EA +12% and SEA +90% vs. the compliant-cell lattice, at lower mass
[69]	Short-CF nylon (Onyx)	Cubic and octet strut lattices; layer-wise topology grading	30%	10 mm cells, 3 × 3 × 3	Quasi-static compression, 10^−3^ s^−1^	NR; NR	Exp. + Num. (FE)	Layered lattices did not absorb more energy than the uniform cubic lattice; cube–octet–cube SEA 19–36% above octet
[66]	PLA core; aluminum face sheets	Honeycomb, chiral and four graded chiral cores; cell-type grading	NR	100 × 100 × 58 mm core	Drop-weight impact, 25 and 50 J (2.94 m/s)	Printed along z; 0.4 mm	Exp.	Graded cores absorbed 98.9–99.8% of the impact energy; the uniform honeycomb had the highest SEA

NR, not reported in the source; no values were estimated. * Nominal strain rate calculated from the reported displacement rate and specimen height. Values are as reported by each study. Because materials, relative densities, specimen sizes and loading conditions differ, entries indicate trends and are not directly comparable. The stereolithography study [59] is discussed in the text as an adjacent comparison and is not tabulated. The full extraction is given in Supplementary Table S1.

**Table 6:** Evidence level of the application claims in Section 7.

Application (Section)	Evidence in the Reviewed Studies	Level
Impact and crash energy absorbers, general (Section 7.1)	Quasi-static and drop-weight tests on graded and multi-material lattice specimens [6,16,36,42,44]	Experimentally demonstrated (specimen level)
Reusable helmet liners, bumper absorbers (Section 7.1)	No prototype; recoverability shown on specimens [16,105]	Prospective
Crash boxes (Section 7.1)	Review of AM crash boxes in general [108]	Prospective
Lattice-filled thin-walled tubes (Section 7.2)	Other process only (laser powder-bed fusion) [109] (adjacent study)	Prospective
Sandwich panels with graded cores (Section 7.2)	Flexural tests on FDM graded sandwich cores [3,106]	Experimentally demonstrated (specimen level)
Aerospace and automotive structural cores (Section 7.2)	No component demonstration	Prospective
Vibration damping (Section 7.3)	Damping capacity measured on TPU lattices filled with PU foam [110]	Experimentally demonstrated (specimen level)
Automotive interiors, precision-equipment mounts (Section 7.3)	No demonstration	Prospective
Transfemoral prosthetic socket (Section 7.4)	PETG/TPU multi-material graded socket fabricated and tested [73]	Prototype-level
Below-knee prosthetic damper (Section 7.4)	Single-material TPU graded lattices, proof-of-concept design [111]	Experimentally demonstrated (specimen level)
Orthopedic insoles (Section 7.4)	Process and density–modulus framework for graded TPE lattices [41]	Experimentally demonstrated (specimen level)
Soft robotics and 4D actuators (Section 7.4)	Dual-material meta-sandwich demonstrators [105]; single-material shape-memory studies [112,113,114]; material jetting [115] (adjacent study)	Experimentally demonstrated (specimen level)
Tissue scaffolds and bioprinting (Section 7.4)	Other processes only [116,117] (adjacent studies)	Prospective

Adjacent study: other process or material, cited for comparison only.

**Table 7:** Comparison of computational methods for MM-FDM graded lattices.

Method	Key Assumptions	Relative Cost	Validation for FDM/MM-FDM Lattices	Main Limitation	Maturity for MM-FDM
Analytical scaling laws	Ideal geometry; isotropic, single-phase parent material	Very low	Used as reference estimates only	Misses FDM anisotropy, defects and interfaces	Established (baseline)
Numerical homogenization (RVE with periodic boundaries)	Scale separation; periodicity; perfect bonding unless modeled	Low–moderate	Process-aware RVE checked against three-point bending of FDM specimens [107]	Discrete multi-material interfaces are costly to homogenize [15] (adjacent study)	Established
Physics-augmented neural-network surrogate	Trained in homogenized data; thermodynamic constraints	High to train, low to use	Numerical only, for resin-printed shell lattices [15] (adjacent study)	Inherits the accuracy limits of its training data	Emerging; not yet applied to MM-FDM
Beam finite elements	Slender struts; nodes not resolved	Low	None reported in the core studies	Misses node stress concentrations [90]	Established (general)
Solid finite elements	Resolved geometry; often perfect bonding	High	Compared with experiments [6,42]	Perfect-bond assumption misrepresents weak dissimilar interfaces [123]	Established
Interface models (cohesive zone, fracture criteria)	Calibrated traction–separation or fracture law	Moderate–high	Calibrated on interfacial fracture tests [6]; mode I/II cracking of IPCs [123,124]	Needs interface-specific calibration data	Emerging
Defect-aware finite elements	Statistical description of as-built geometry	High	Process-induced anisotropy included and checked experimentally [107]; [21,89]	Needs micro-CT or metrology input	Emerging
Implicit, voxel and direct G-code workflows	Field-defined geometry or material maps	Low–moderate	Printed graded demonstrations [81,84]; [126]	Slicers still discretize gradients stepwise	Established (design side)
Topology optimization (SIMP, multi-material)	Density interpolation; linear-elastic objectives	Moderate–high	Flexural FDM specimens, non-lattice [128]	Few formulations include switching, purge or interface-strength constraints	Limited for MM-FDM
Surrogate machine learning with multi-objective search	Training data span the design space	Moderate	Checked against printed PLA/TPU lattices [16,93] and dual-material FFF composites [130]	Unreliable outside the training range	Emerging
Generative models (GNN, latent diffusion)	Learned design distributions	High	None for MM-FDM graded lattices; [122] numerical, [121] single-material	No manufacturability constraints or MM-FDM validation	Proposed

Citations after a semicolon are supporting studies; adjacent studies (other process) are cited for comparison only.

## Data Availability

No new experimental data were generated. The merged search records (BibTeX), the screening script with its outputs, the scoring rubric, the record-level screening and classification table (including retrospective full-text exclusion reasons), and the bibliometric script and data are provided as Supplementary Materials S1–S4 and are openly available at Zenodo: https://doi.org/10.5281/zenodo.23156671.

## Supplementary Materials

The following supporting information can be downloaded at https://doi.org/10.5281/zenodo.23156671. Table S1: Record-level screening and classification of the search records and references; Table S2: Stage 1 keyword-scoring rubric; Material S3: merged search records, screening script and screening outputs; Material S4: bibliometric scripts, input data and outputs for Figure 3 and Figure 4 can be found in Zenodo: https://doi.org/10.5281/zenodo.23156671.

## Abbreviations

ABS	acrylonitrile butadiene styrene
AM	additive manufacturing
BCC	body-centered cubic
CAD	computer-aided design
CTE	coefficient of thermal expansion
CZM	cohesive zone model
DfAM	design for additive manufacturing
DIC	digital image correlation
FCC	face-centered cubic
FDM	fused deposition modeling
FEA	finite element analysis
FFF	fused filament fabrication
FGF	fused granular fabrication
FGL	functionally graded lattice
FGM	functionally graded material
GNN	graph neural network
IDEX	independent dual extrusion
IPC	interpenetrating phase composite
LDM	latent diffusion model
MEX	material extrusion
ML	machine learning
MM-FDM	multi-material fused deposition modeling
MMAM	multi-material additive manufacturing
PEI	polyetherimide
PETG	polyethylene terephthalate glycol
PLA	polylactic acid
RVE	representative volume element
SEA	specific energy absorption
SIMP	solid isotropic material with penalization
SMP	shape memory polymer
TO	topology optimization
TPMS	triply periodic minimal surface
TPU	thermoplastic polyurethane
μ-CT	micro-computed tomography
